# Antimicrobial Potency and *E. coli* β-Carbonic Anhydrase Inhibition Efficacy of Phenazone-Based Molecules

**DOI:** 10.3390/molecules28227491

**Published:** 2023-11-08

**Authors:** Huda R. M. Rashdan, Gharieb S. El-Sayyad, Ihsan A. Shehadi, Aboubakr H. Abdelmonsef

**Affiliations:** 1 Chemistry of Natural and Microbial Products Department, Pharmaceutical and Drug Industries Research Institute, National Research Centre, 33 El Buhouth St., Dokki, Giza 12622, Egypt; 2 Department of Microbiology and Immunology, Faculty of Pharmacy, Ahram Canadian University (ACU), Giza 12566, Egypt; gharieb.elsayyad@acu.edu.eg; 3 Department of Microbiology and Immunology, Faculty of Pharmacy, Galala University, New Galala City, Suez 43511, Egypt; 4 Drug Microbiology Laboratory, Drug Radiation Research Department, National Center for Radiation Research and Technology (NCRRT), Egyptian Atomic Energy Authority (EAEA), Cairo 11765, Egypt; 5 Chemistry Department, College of Sciences, University of Sharjah, Sharjah 27272, United Arab Emirates; 6 Chemistry Department, Faculty of Science, South Valley University, Qena 83523, Egypt; aboubakr.ahmed@sci.svu.edu.eg

**Keywords:** phenazone, methyl hydrazinecarbodithioate, antimicrobial potency, molecular docking

## Abstract

In this investigation, 4-antipyrinecarboxaldhyde was reacted with methyl hydrazinecarbodithioate to afford the carbodithioate derivative **3**. The as-prepared carbodithioate derivative **3** is considered to be a key molecule for the preparation of new antipyrine-1,3,4-thiadiazole-based molecules (**4**–**9**) through its reaction with the appropriate hydrazonoyl halides. Furthermore, a typical Biginelli three-component cyclocondensation reaction involving ethyl acetoacetate, 4-antipyrinecarboxaldhyde, and thiourea under the standard conditions is carried out in the presence of sulfuric acid to afford the corresponding antipyrine–pyrimidine hybrid molecule (**10**). The latter was submitted to react with hydrazine monohydrate to provide the corresponding hydrazide derivative (**11**) which, under reaction with ethyl acetoacetate in refluxing ethanol containing catalytic amount of acetic acid, afforded the corresponding derivative (**12**). The structure of the newly synthesized compounds was affirmed by their spectral and microanalytical data. We also screened for their antimicrobial potential (ZOI and MIC) and conducted a kinetic study. Additionally, the mechanism of biological action was assessed by a membrane leakage assay and SEM imaging technique. Moreover, the biological activities and the binding modes of these compounds were further supplemented by an in silico docking study against *E. coli* β-carbonic anhydrase. The amount of cellular protein released by *E. coli* is directly correlated to the concentration of compound **9**, which was found to be 177.99 µg/mL following treatment with 1.0 mg/mL of compound **9**. This finding supports compound **9**’s antibacterial properties and explains how the formation of holes in the *E. coli* cell membrane results in the release of proteins from the cytoplasm. The newly synthesized compounds represent acceptable antimicrobial activities with potential action against *E. coli* β-carbonic anhydrase. The docking studies and antimicrobial activity test proved that compound (**9**) declared a greater activity than the other synthesized compounds.

## 1. Introduction

**Figure 1:**
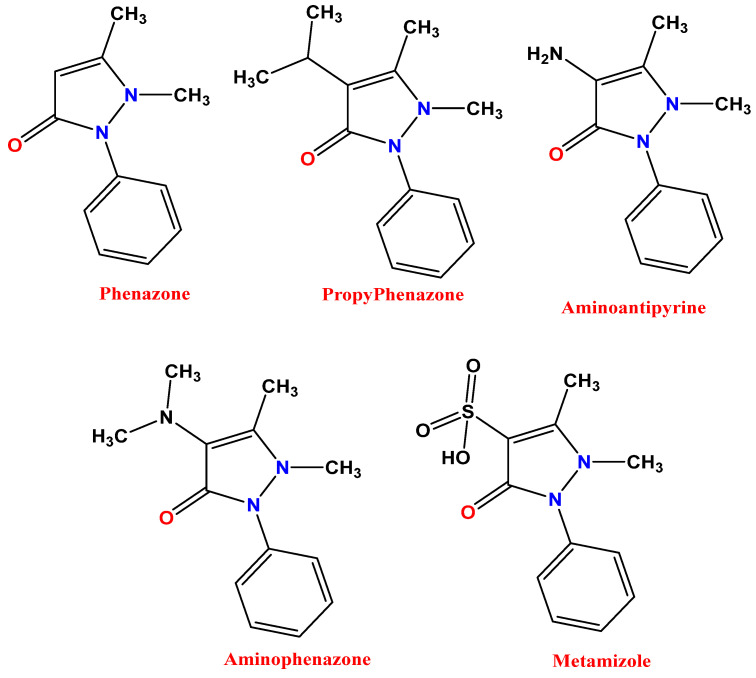
Chemical structures of some drugs based on antipyrine.

Antipyrine-based molecules continue to be of great interest for both medicinal chemists and organic chemists owing to their richness in synthetic compounds with multifarious involvement in pharmaceutical and medicinal compounds [1,2]. In the last decade, they have been considered to be very important compounds because of their biomedical and pharmaceutical applications. In this regard, antipyrine has been integrated in different heterocyclic derivatives. Additionally, their synthesis strategies continue today. In various clinical, biomedical, and pharmaceutical applications, antipyrine-based molecules are reported likewise to have anti-inflammatory, anticancer [3,4], analgesic [5], antipyretic [4], antimicrobial [6,7], etc., effects. Meanwhile, antipyrine is the core structure often found in many different drugs such as Metamizole, which is known commercially as Novalgine, Propyphenazone, Aminophenazone, Aminoantipyrine, and Phenazone (Figure 1).

On the other hand, 1,3,4-thiadiazoles [8,9,10,11,12,13,14,15,16,17] and their analogies have revealed a wide variety of effects, such as antiviral, anti-inflammatory, antidiabetic, anticancer, antimicrobial, and anticonvulsant ones, in biomedical and pharmaceutical applications [18,19]. Moreover, pyrimidines and their hybrid molecules are considered to be a favorable research area for medicinal chemists [20,21,22,23,24,25,26,27,28,29,30] owing to their richness in both synthetic and natural bioactive molecules along with various involvement in polymers, biopolymers, and pharmaceuticals, and they can act as ligands. Consequently, refining linker conjugation between antipyrine and the other bioactive heterocycle derivatives is expected to boost their potential activities [31,32,33,34,35,36,37,38].

Increased morbidity and mortality are caused by specific types of *E. coli*, particularly among immunocompromised individuals utilizing urethral and intravascular catheters [39]. The formation of biofilms makes treating infections brought on by *E. coli* difficult.

These biofilms are composed of bacterial colonies encased in an extracellular polymeric substance (EPS) matrix that protects germs from unfavorable environmental factors that may otherwise result in infection. *E. coli* biofilm causes intrinsic medical-device-related infectivity in addition to causing recurrent urinary tract infections [40]. Antibiotic-resistant cells develop as a result of biofilm, which decreases the spread of standard antibiotics [40]. By changing the target enzymes, lessening the cell’s permeability to prevent its entrance, or actively transporting the medication out of the cell, *E. coli* can develop resistance to antibiotics [41]. Antibiotic resistance can result from any of these resistance mechanisms, although target site mutations seem to be the most significant.

It is important to look for alternative therapeutic agents since biofilm is a target in the battle against bacterial illnesses that are drug-resistant.

In this report, and in continuation of our previous work on the synthesis of novel bioactive compounds, a new series of thiadiazole and pyrimidine moieties attached to an antipyrine analogue was synthesized via conventional methods. In addition, their antimicrobial activities were evaluated through in vitro and in silico studies.

## 2. Results and Discussion

### 2.1. Chemistry

**Scheme 1:**
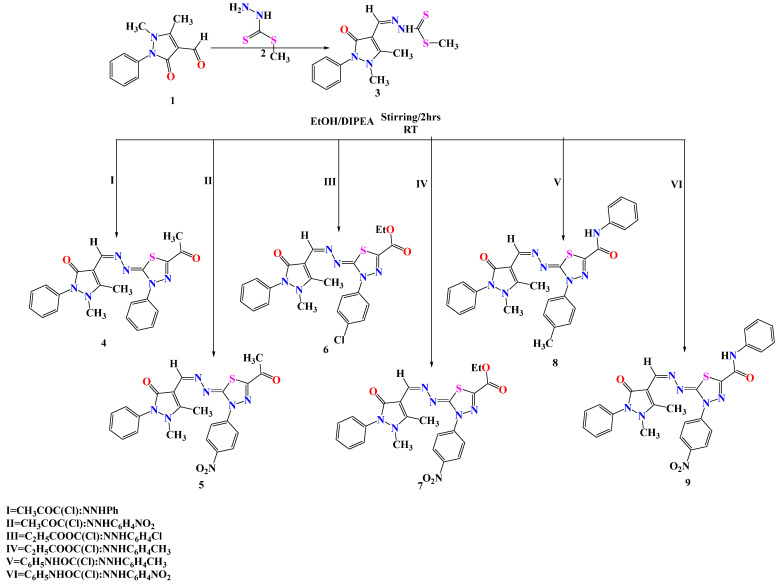
Synthetic procedures of antipyrine–thiadiazole hybrids **3–9**.

4-antipyrinecarboxaldhyde (**1**) was allowed to react with methylhydrazine carbodithioate (**2**) to afford methyl-2-((1,5-dimethyl-3-oxo-2-phenyl-2,3-dihydro-1*H*-pyrazol-4-yl) methylene) hydrazine-1-carbodithioate (**3**) [42]. We were also interested in architecting new 1,3,4-thiadiazole-based molecules by incorporating antipyrine conjugates which were generated via the reaction of **3** with the appropriate hydrazonoyl halides in absolute ethanol containing catalytic amounts of DIPEA at room temperature (Scheme 1). The chemical structure of the newly synthesized derivatives was affirmed by spectral data. For instance, the ^1^H-NMR spectrum of compound **4** revealed significant signals at 2.42 and 2.56 ppm for the 6 protons of 2 methyl groups, along with a signal at 3.22 ppm attributed to the three protons of the N-CH_3_ group. It also exhibited a multiplet signal at 7.27–7.7.96 ppm for the (CH) proton. Additionally, the ^13^C-NMR spectrum showed characteristic signals at 12.34 and 24.28 for the two methyl carbons. Also, it revealed a significant signal at 34.21 ppm for the (N-**CH_3_**) carbon, with 94.12, 121.32, 121.72, 122.50, 123.70, 123.88, 125.82, 127.82, 129.26, 132.82, 133.12, 134.22, 134.82, and 151.53 ppm being assigned for the aromatic carbons. Additionally, it showed signals at 158.18 ppm for (C=N), 163.7 ppm for (H-C=N), 164.41 ppm for (C=O), and 195.81 ppm for (C=O). The structure was supported also by its mass spectrum *m*/*z* (%): 432, which is in agreement with its molecular formula C_22_H_20_N_6_O_2_S. Meanwhile, the chemical structure of compound **6** was secured based on its correct spectral analyses. Its FT-IR spectrum revealed a strong absorption band at 1715 cm^−1^ represented the carbonyl ester. Its ^1^H-NMR spectrum displayed a significant triplet signal at *δ* 1.31 ppm for the protons of the methyl group of (CH_2_**CH_3_**), a singlet signal at 2.63 ppm for the three protons of the methyl group, a singlet signal at 3.30 ppm for the proton of the methyl group of (N-**CH_3_**), and a doublet signal at 4.36 ppm for the protons of CH_2_ of (**CH_2_**CH_3_). In addition, the aromatic protons recorded a multiplet signal in the region from *δ* 7.34 to 7.60 ppm. Also, a singlet signal at 8.19 ppm was shown for the proton of (CH). Its ^13^C-NMR spectrum demonstrated significant signals at *δ* 12.33 ppm for (CH_3_), 12.35 ppm for (CH_2_**CH_3_**), 13.93 ppm for (N-**CH_3_**), 62.77 ppm for (**CH_2_**CH_3_), 163.71 ppm for (C=O), and 168.72 ppm attributed to (C=O). The mass spectrum exhibited a molecular ion peak at *m*/*z* = 496 (M^+^) and isotopic peak [M^+^ + 2] at *m*/*z* = 498 in an approximate ratio of 1:1, which supported the molecular formula (C_23_H_21_ClN_6_O_3_S).

**Scheme 2:**
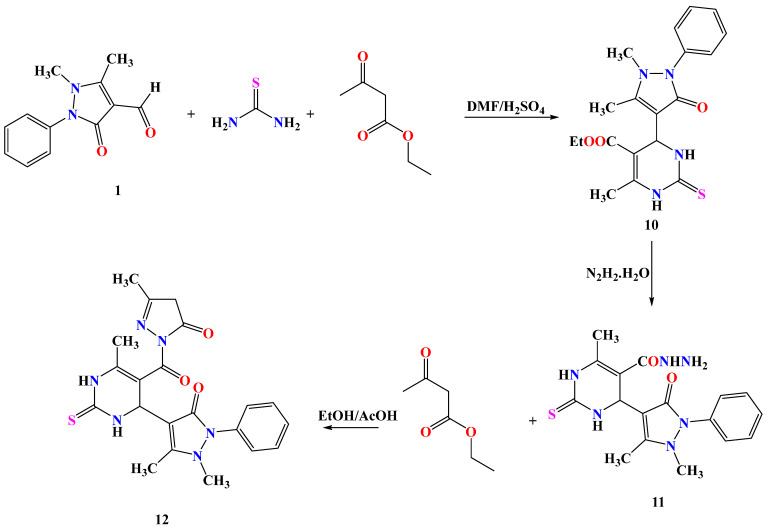
Synthetic procedures of antipyrine–pyrimidine hybrids **10**–**12**.

Additionally, a new polysubstituted pyrimidine derivative containing antipyrine moiety was constructed via the Biginelli three-component cyclocondensation reaction [43,44,45,46] of ethyl acetoacetate, 4-antipyrinecarboxaldhyde (**1**), and thiourea to afford the desired molecule **10**. In the reaction performed via the tetrahedral mechanism, in which the N-C bond was formed first and then the C-O bond begin to break, the elimination of ethanol occurred, and so a lot of energy was accumulated in the medium of the reaction to offset the activation energy of the reaction, and a conversion took place, followed by cyclization via the nucleophilic attack through the amine group onto the carbonyl group to give the target molecule **10**. Compound **10** was reacted with hydrazine hydrate to yield the corresponding hydrazide derivative **11**, which then reacted with ethyl acetoacetate in ethanol containing a catalytic amount of acetic acid to afford the corresponding pyrazole derivative **12** [47] (Scheme 2). The chemical structure of **12** was confirmed based on correct spectral data, in which its ^1^H-NMR spectrum revealed three singlet signals at *δ* 1.94, 2.26, 3.34 ppm for three methyl groups, a doublet signal at *δ* 3.12 ppm for protons of (CH_2_-pyrazole), a singlet signal at *δ* 3.95 ppm for the proton of (CH-4, pyrimidine), a multiplet signal in the region from *δ* 7.32 to 7.52 ppm for the aromatic protons, and two singlet signals at *δ* 8.25 and 10.01 ppm for the NH protons. Its ^13^C-NMR spectrum showed significant signals at *δ* 15.72 ppm for (CH_3_), 17.94 ppm for (CH_3_), 42.15 ppm for (CH_2_-pyrazole), 51.22 ppm for (CH-4, pyrimidine), 106.14, 108.24, 120.12, 121.61, 122.12, 123.01, 134.57, 143.09, 152.98, and 159.14 ppm, which represented the aromatic carbons, 163.24 ppm for (C=O), 164.58 ppm for (C=O), 165.19 ppm for (C=O), and 182.12 ppm for (C=S).

### 2.2. Antimicrobial Activity of the Newly Synthesized Compounds

**Figure 2:**
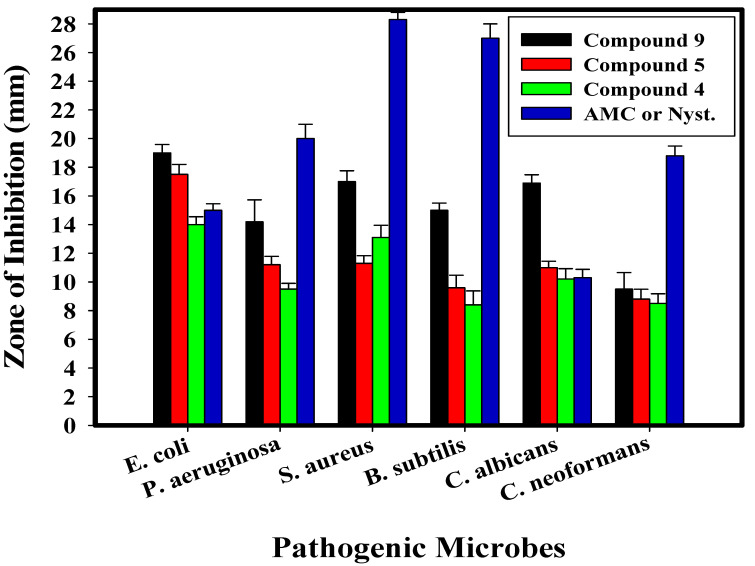
Antimicrobial activity of the synthesized compounds against different bacterial and fungal strains as IZ.

The antibacterial potential of each produced compound was examined; compounds **4**, **5**, and **9** exhibited the greatest levels of activity. According to Table 1 and Figure 2, the antimicrobial efficacy of compounds **4**, **5**, and **9** against various bacterial and fungal strains was assessed. In comparison to AMC/Nyst, which are common antimicrobial agents, all the developed compounds showed promising antibacterial activity against all of the evaluated bacterial and fungal species. Compared to AMC/Nyst, compounds **4**, **5**, and **9** were noticeably more active.

The results showed that compound **9**, along with compounds **4** and **5**, has the greatest impact on the majority of examined bacterial and fungal strains. According to Table 1, of all the investigated bacterial strains, compound **9** (100 µg/mL; 100 ppm) had the greatest effect on *E. coli*. With inhibition zones of 16.9 mm and 19.0 mm, it had the strongest effect on *C. albicans* of all the studied fungal strains. Additionally, compound **9** showed potential antibacterial action against *E. coli*, *P. aeruginosa*, *S. aureus*, *B. subtilis*, *C. albicans*, and *C. neoformans*, with inhibition zones of 19.0, 14.2, 17.0, 15.0, and 16.9 mm, respectively, at a concentration of 100 µg/mL (100 ppm). The largest effect was against *E. coli* and *S. aureus* with inhibition zones of 14.0 and 13.1 mm, respectively, for compound **4** and 17.5 and 11.3 mm for compound **5** against *E. coli* and *B. subtilis*. However, compounds **4** and **5** possessed antibacterial activity that was less potent than compound **9**. Additionally, the MICs of every sample that was evaluated (compounds **4**, **5**, and **9**) were established, as shown in Table 1.

The results showed that, when compared to other examined microbiological strains, compounds **9**, **5**, and **4** had the best MICs towards the tested bacteria and unicellular fungus, with MICs in the range of 15.62–500 µg/mL. Additionally, *E. coli* was shown to be the most susceptible of the studied bacteria, with the MICs of compounds **9**, **5**, and **4** being 15.62, 31.25, and 125 µg/mL, respectively. On the other hand, the MIC of every substance against *E. coli*, *P. aeruginosa*, and *S. aureus* ranged from 31.25 to 500 µg/mL, which was lower than that of *B. subtilis*.

Finally, compared to standard antimicrobial drugs (AMC/Nyst), the proposed compounds showed good antibacterial efficacy against some bacterial isolates (*E. coli*) and unicellular fungi (*C. albicans*). On the other hand, the utilized standard antibiotics (AMC/Nyst) were more active than the synthesized compound against some tested microbes due to the fact that the concentration used in our synthesized compound **9** (15.62 µg/mL; MIC result in Table 1) was less than that recoded in the applied standard antibiotics (AMC/Nyst) to avoid toxicity and utilize the synthesized compound **9** in a low concentration with elevated antimicrobial activity.

### 2.3. Kinetics of E. coli Growth (Growth Curve)

**Figure 3:**
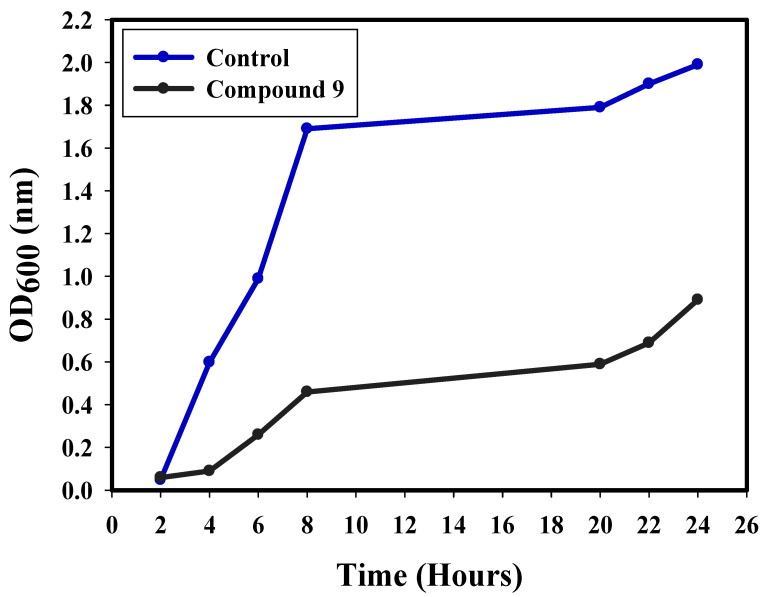
Effect of compound **9** on the kinetics of *E. coli* growth with time intervals (h).

The influence of compound **9** on the growth kinetics of *E. coli* cells was investigated. Figure 3 depicts how quickly *E. coli* seemed to develop in the control sample. The control sample’s O.D. at 600 nm is 1.99. Due to compound **9**’s remarkable antibacterial effect, the OD_600_ values of the treated cells are lower than those of the control sample. 

From the beginning of the observation to the endpoint of 24 h (O.D. 0.89), compound **9** treatment results in a rate of bacterial growth suppression. At the start of the observation, there is no discernible difference in the effects of compound **9**’s concentrations. In addition, as shown by the O.D. data (Figure 3), compound **9** has a stronger inhibitory effect than the control. When the *E. coli* growth rates without compound **9** are higher than the growth rates with compound **9**, the consequences are said to have occurred. A substance must stick to its target sites on the microbial cells and settle in a specific number of crucial regions associated to its concentration within the pathogenic microorganisms in order for it to have antimicrobial potentials that can kill those germs. 

### 2.4. Determination of Protein Leakage from Bacterial Cell Membranes

**Figure 4:**
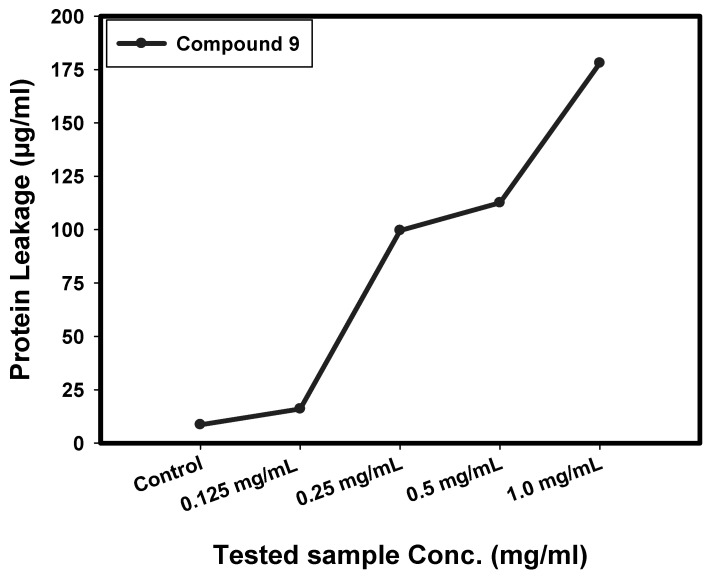
The effect of compound **9** on the protein leakage from *E. coli* cell membranes.

The Bradford technique was used to calculate the amounts of protein released in the suspension of the treated *E. coli* bacteria. According to Figure 4, the amount of cellular protein released by *E. coli* is directly correlated to the concentration of compound **9**, which was found to be 177.99 µg/mL following the treatment of compound **9** with 1.0 mg/mL. This finding supports compound **9**’s antibacterial properties and explains how the formation of holes in the *E. coli* cell membrane results in the release of proteins from the cytoplasm.

According to these test results, compound **9** made *E. coli* cell membranes more permeable; therefore, it was plausible to anticipate that membrane permeability would be mistaken for an essential factor in the reduction in bacterial mass. 

Similar results were found when ferrites were used, as described in related studies, which showed concentration-dependent membrane instability in bacterial cells and indicated intracellular substance leakage (bacterial cell suspension).

Paul et al. [48] demonstrated that the percentage difference in the corresponding electric conductivity corresponded to the difference in bacterial cell membrane permeability. According to various reports, when the quantity of the treated compounds increased, the proportion of the tested samples’ relative electric conductivities also increased. A study of the discharge of bacteria’s cell components, such as proteins, was used to determine the integrity of the bacterial cell membrane. The leakage progressed over time due to persistent cell membrane damage, which suggested that the discharge of cell components was what ultimately led to cell death.

### 2.5. Mechanism of Biological Action Determination by SEM

**Figure 5:**
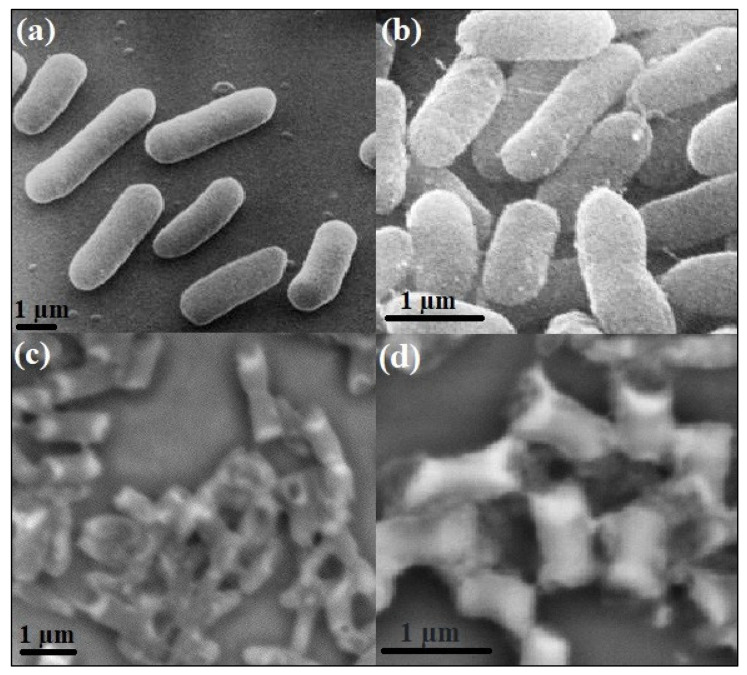
Mechanism of biological action determination of compound **9** using SEM analysis, with (**a**,**b**) control untreated *E. coli* and (**c**,**d**) treated *E. coli*.

Figure 5 shows how SEM analysis was used to show a potential antimicrobial behavior against *E. coli*. As shown in Figure 5a,b, the SEM investigation of the control bacterial cells in the absence of compound **9** revealed bacterial groups that generally prolonged and grew with a regular surface, a normal shape, and a normal count.

After having been subjected to compound **9** (the most active compound), *E. coli* displayed striking morphological abnormalities (Figure 5c,d), which included semi-lysis of the outermost layer in certain bacterial cells brought on by deformations of the *E. coli* cells. Furthermore, the synthetic substance **9** entirely damaged bacterial cells and caused deformities of them, leading to a decrease in the number of cells that survived overall (Figure 5c,d), and created holes on the surface of bacterial cells. The membrane leakage experiment demonstrated that coating was produced over the bacterial cells as a result of covalent bond formation due to chemisorption attractions between compound **9** and the bacterial cell.

The synthesized Se NPs-gentamicin (CN) nano-drug, on the other hand, was subjected to SEM imaging against *E. coli*, describing the antibacterial response mechanism after discovering that *E. coli* cells exhibited morphological alterations following treatment with Se NPs-CN. Bacterial cell deformity and a discernible increase in the hardness of the bacterial cell surface indicated that Se NPs-CN was suppressing and controlling it. They were also lower in number, and the biofilm was impeded.

### 2.6. Molecular Docking Studies and ADMET Analysis

**Figure 6:**
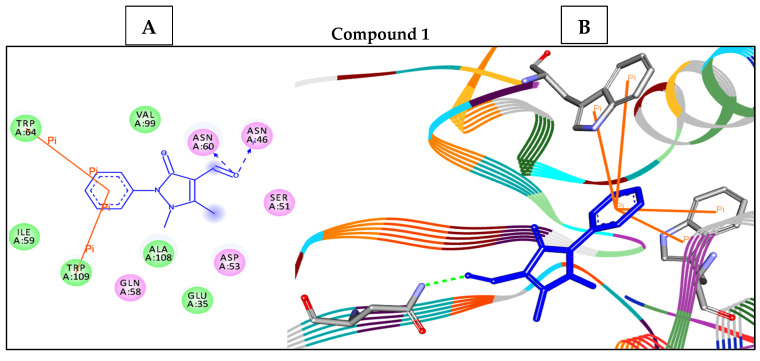

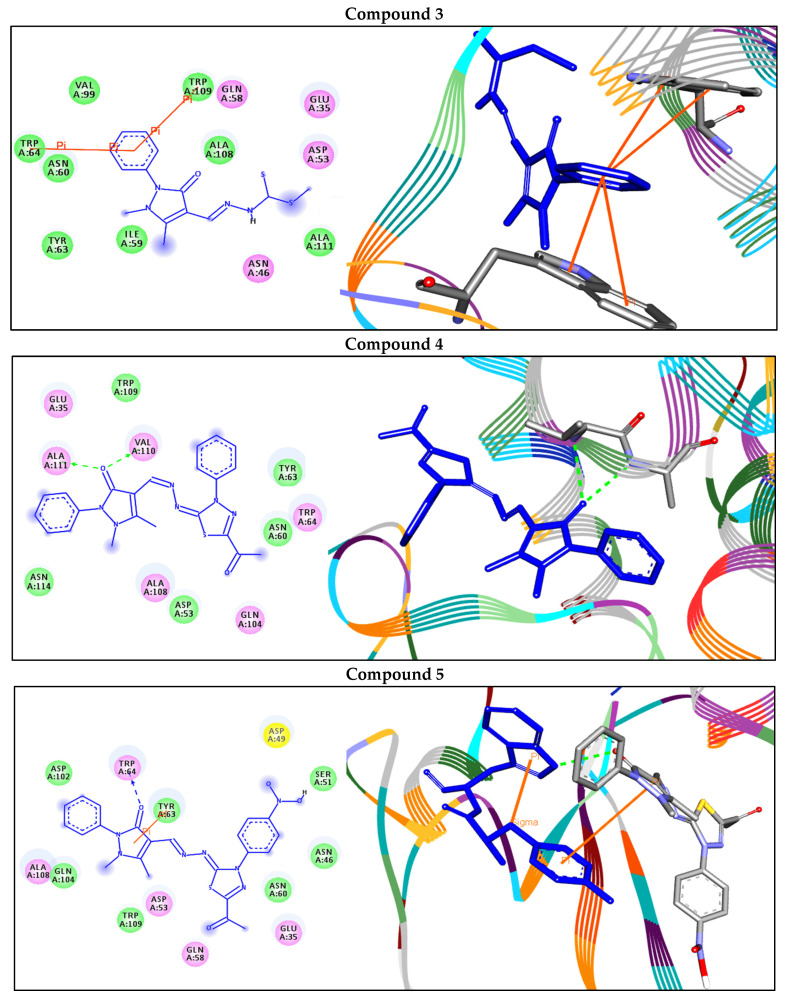

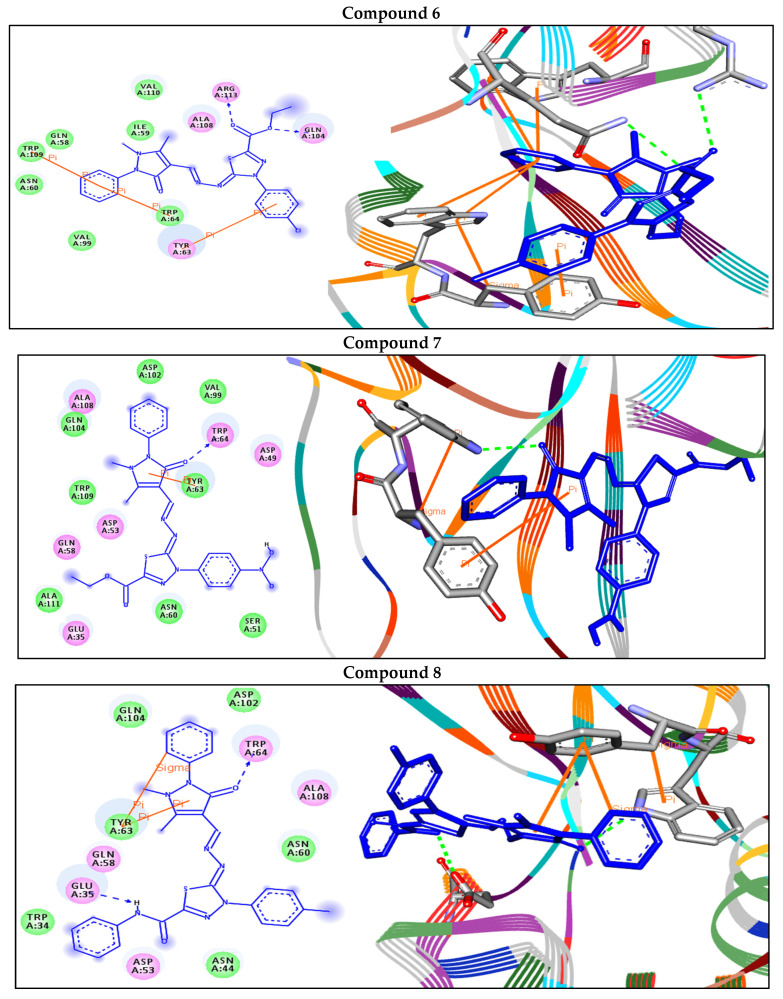

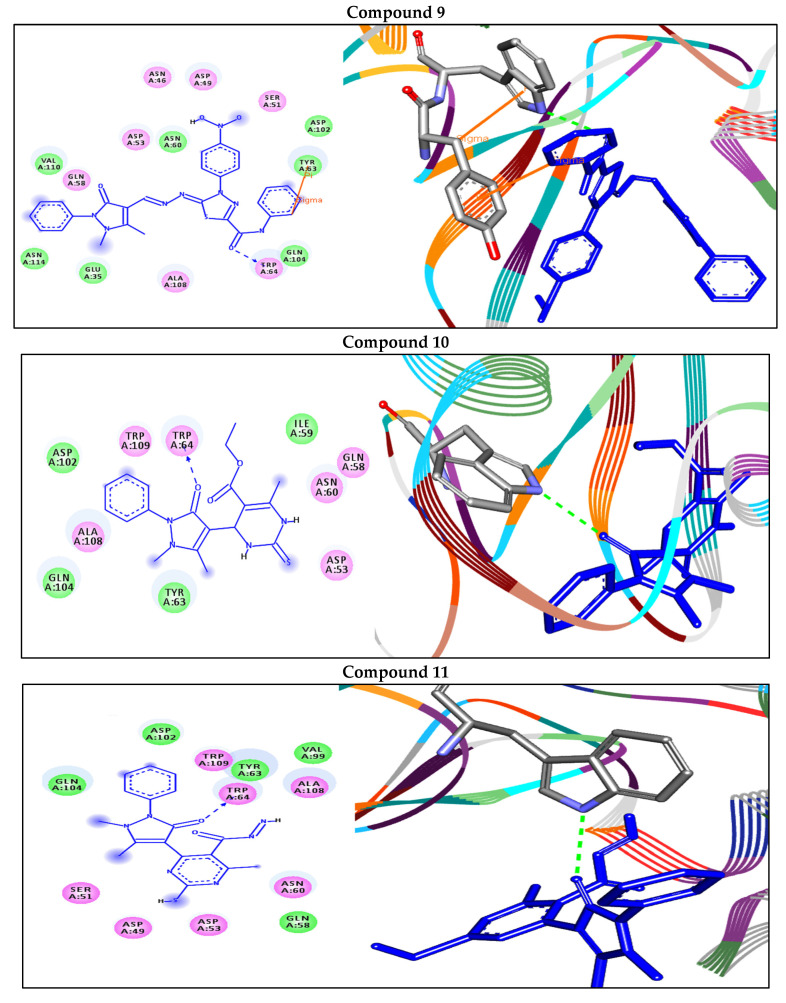

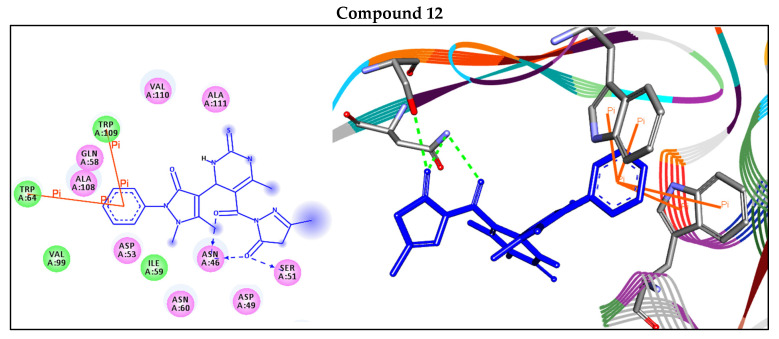
Docking results of the synthesized compounds against the target *E. coli* β-carbonic anhydrase. (**A**) Two-dimensional (2D) and (**B**) three-dimensional (3D) orientations of docked complexes. H-bond interactions are represented in blue and green dotted lines. Pi-stacked interactions are shown in orange lines.

**Figure 7:**
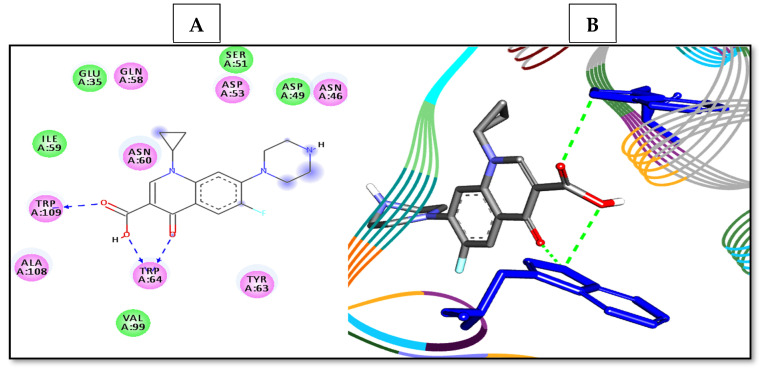
Docking results of reference drug against *E. coli* β-carbonic anhydrase. (**A**) Two-dimensional (2D) and (**B**) three-dimensional (3D) orientations of docked complexes. H-bond interactions are represented in blue and green dotted lines. Pi-stacked interactions are shown in orange lines.

Carbonic anhydrases CAs are important metalloenzymes widely present in all living organisms and catalyze the essential reaction of CO_2_ hydration. In addition, they play a pivotal role in many cellular and physiological processes. In the present study, an in silico docking technique is used to investigate the binding mode of actions of the newly synthesized compounds and a reference drug against the target enzyme *E. coli* β-carbonic anhydrase. From the data obtained, the newly compounds displayed respectable fitting to the active site residues of the enzyme, which showed binding energies ranging from −9.7 to −7.0 kcal/mol. Compound **9** unveiled the best binding energy (−9.7 kcal/mol) and showed H-bond and arene–sigma interactions with the residues TRP64 and TYR63, respectively. On the other hand, compound **3** showed the lowest binding energy (−7.0 kcal/mol) against the target enzyme, as represented in Table 2 and Figure 6. The reference drug also docked similarly to the residues through H-bond interactions (Figure 7). 

**Figure 8:**
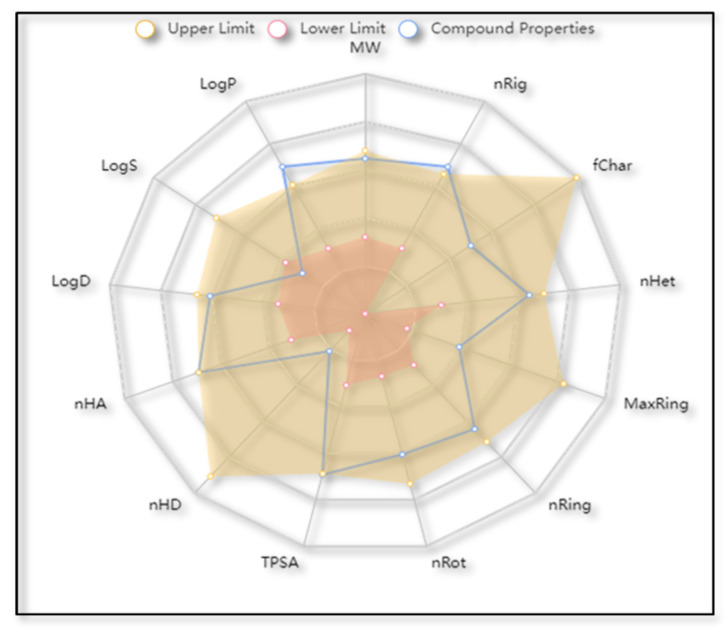
ADMET analysis of compound **9** calculated by ADMET lab tool.

On the other hand, Table 3 represents the physicochemical and pharmacokinetics properties of the compounds. The bioavailability and physicochemical parameters of the best docked compound **9**, as an example, were calculated using the ADMET lab tool, by plotting radar showing 13 properties (Figure 8). All compounds except compound **1** did not pass the blood–brain barrier, indicating that they have a good CNS safety profile. The compounds showed no Lipinski violation, confirming the ability to use these compounds as antibacterial candidates.

## 3. Materials and Methods

### 3.1. Chemistry

#### 3.1.1. Raw Materials

4-antipyrinecarboxaldehyde, methyl hydrazinecarbodithioate, ethyl acetoacetate 99%, thiourea, and hydrazine hydrate 99% were procured from Sigma-Aldrich. Some reagents and solvents were used in this study like DMF, diisopropylethylamine, absolute ethanol, isopropyl alcohol 99%, and acetic acid 99.9% were imported also from Sigma-Aldrich, Milwaukee, WI, USA. All solvents and chemicals were used without further purification.

#### 3.1.2. Instrumentation

All melting points were uncorrected and measured using an electrothermal device. The IR spectra were recorded (KBr discs) using the Shimadzu FT-IR 8201 PC spectrophotometer (Kyoto, Japan). ^1^H- and ^13^C-NMR spectra were recorded in (CD_3_)_2_SO solutions on a BRUKER 500 FT-NMR system spectrometer (Billerica, MA, USA), and chemical shifts were expressed in ppm units using TMS as an internal reference. Mass spectra were recorded on a GC-MS QP1000 EX Shimadzu (Kyoto, Japan). Elemental analyses were carried out at the Microanalytical Center of Cairo University, Egypt.

#### 3.1.3. Synthetic Procedures of the Target Molecules

##### Synthetic Procedures of the Target Molecule methyl-2-((1,5-dimethyl-3-oxo-2-phenyl-2,3-dihydro-1H-pyrazol-4-yl)methylene)hydrazine-1-carbodithioate (**3**)

A mixture of 4-antipyrinecarboxaldhyde **1** (1.08 g, 5 mmol) and the methyl hydrazinecarbodithioate **2** (0.6 g, 5 mmol) in 20 mL isopropyl alcohol was stirred at room temperature for 2 h, and the reaction was monitored by TLC. The solid was collected and washed with (water/ethanol) the recrystallized from the ethanol to give the desired derivative. Yellow crystals (85%); m.p.: 200–202 °C, FT-IR (KBr, cm^−1^): *v* 3296 (N-H), 1664 (N-C=O), 1620 (C=N), 1595 (C=C); ^1^H-NMR (DMSO-d*_6_*): *δ* 2.47 (s, 3H, CH_3_), 2.60 (s, 3H, S-CH_3_), 3.26 (s, 3H, N-CH_3_), 7.30–7.51 (m, 5H, Ar-H), 8.05 (s, 1H, CH), 13.03 (s, 1H, NH);^13^C-NMR (100 MHz, DMSO-d*_6_*): *δ* 12.22 (CH_3_), 18.48 (S-CH_3_), 34.09 (N-CH_3_), 123.70 (Ar), 123.88 (Ar), 125.82 (Ar), 127.82 (Ar), 129.26 (Ar), 132.82(Ar), 151.53(Ar), 155.18(CH), 164.41(C=O), 195.81 (C=S); MS *m*/*z* (%): 320 (M^+^, 60). Anal. Calcd. for “C_14_H_16_N_4_OS_2_” (320): C, 52.48; H, 5.03; N, 17.49 Found: C, 52.42; H, 5.01; N, 17.43%.

##### Synthetic Procedures of the Target Molecules (**4**–**9**)

A mixture of compound **3** (1.6 g, 5 mmol) and the selected derivatives of the hydrazonoyl halides (5 mmol) and 2–3 drops of diisopropylethylamine (DIPEA) as a catalyst in 10 mL ethanol was stirred at room temperature for 2 h. The stirring continued for approximately 5 h, and the reaction was monitored by TLC. The solid was collected and washed with (water/ethanol) the recrystallized from the proper solvent to give the desired derivatives **4**–**9**, respectively.

*4-(5-acetyl-3-phenyl-1,3,4-thiadiazol-2(3H)-ylidene)hydrazono)methyl)-1,5-dimethyl-2-phenyl-1,2-dihydro-3H-pyrazol-3-one* (**4**), yellow crystals from acetic acid (78%); m.p. 260–261 °C, FT-IR (KBr, cm^−1^): 1684 (C=O), 1675 (N-C=O), 1610 (C=N), 1580 (C=C); ^1^H-NMR (DMSO-d*_6_*): *δ* 2.42 (s, 3H, CH_3_), 2.56 (s, 3H, CH_3_), 3.22 (s, 3H, N-CH_3_), 7.27–7.7.96 (m, 10H, Ar-H), 8.09 (s, 1H, CH); ^13^C-NMR (100 MHz, DMSO-d*_6_*): *δ* 12.34 (CH_3_), 24.28 (CH_3_), 34.21 (N-CH_3_), 94.12 (Ar), 121.32 (Ar), 121.72 (Ar), 122.50 (Ar), 123.70 (Ar), 123.88 (Ar), 125.82 (Ar), 127.82 (Ar), 129.26 (Ar), 132.82(Ar), 133.12(Ar), 134.22 (Ar), 134.82 (Ar), 151.53 (Ar), 158.18 (C=N), 163.7 (H-C=N), 164.41 (C=O), 195.81 (C=O); MS *m*/*z* (%): 432 (M^+^, 22). Anal. Calcd. for “C_22_H_20_N_6_O_2_S” (432): C, 61.10; H, 4.66; N, 19.43; Found: C, 61.12; H, 4.61; N, 19.38%.

*4-(5-acetyl-3-(4-nitrophenyl)-1,3,4-thiadiazol-2(3H)-ylidene)hydrazono)methyl)-1,5-dimethyl-2-phenyl-1,2-dihydro-3H-pyrazol-3-one* (**5**), yellowish brown crystals from acetic acid (62%); m.p. 245–247 °C, FT-IR (KBr, cm^−1^): 1686 (C=O), 1672 (N-C = O), 1600 (C=N), 1580 (C=C); ^1^H-NMR (DMSO-d_6_): δ 2.36 (s, 3H, CH_3_), 2.48 (s, 3H, CH_3_), 3.22 (s, 3H, N-CH_3_), 7.06–7.47 (m, 9H, Ar-H), 7.94 (s, 1H, CH); ^13^C-NMR (100 MHz, DMSO-d_6_): δ 12.36 (CH_3_), 24.18(CH_3_), 34.22(N-CH_3_), 95.22 (Ar), 120.82 (Ar), 122.72 (Ar), 122.98 (Ar), 123.78 (Ar), 123.18(Ar), 124.62 (Ar), 128.52 (Ar), 129.16 (Ar), 132.52 (Ar), 133.22 (Ar), 134.12 (Ar), 134.82 (Ar), 151.23 (Ar), 158.18 (C=N), 163.18 (H-C=N), 163.41 (C=O), 197.81(C=O); MS *m*/*z* (%): 477 (M^+^, 51). Anal. Calcd. for “C_22_H_19_N_7_O_4_S” (477): C, 55.34; H, 4.01; N, 20.53; Found: C, 55.29; H, 3.97; N, 20.46%.

*Ethyl 4-(4-chlorophenyl)-5-(1,5-dimethyl-3-oxo-2-phenyl-2,3-dihydro-1H-pyrazol-4-yl)methylene)hydrazono)-4,5-dihydro-1,3,4-thiadiazole-2-carboxylate* (**6**), yellow crystals from acetic acid (82%); m.p. 175–177 °C, FT-IR (KBr, cm^−1^): 1675 (N-C = O), 1715(C=O), 1615 (C=N), 1600 (C=C); ^1^H-NMR (DMSO-d_6_): δ 1.31(t, 3H, CH_2_CH_3_), 2.63 (s, 3H, CH_3_), 3.30 (s, 3H, N-CH_3_), 4.36(d, 2H, CH_2_CH_3_), 7.34–7.60 (m, 9H, Ar-H), 8.19 (s, 1H, CH); ^13^C-NMR (100 MHz, DMSO-d_6_): δ 12.33 (CH_3_), 12.35 (CH_2_CH_3_), 13.93 (N-CH_3_), 62.77 (CH_2_CH_3_), 86.40 (Ar), 99.75 (Ar), 123.25 (Ar), 125.78 (Ar), 127.67(Ar), 129.00(Ar), 129.19 (Ar), 133.19 (Ar), 134.48 (Ar), 138.20 (Ar), 149.95 (Ar), 152.86 (Ar), 158.30(Ar), 163.30 (CH), 163.71 (C=O), 168.72 (C=O); MS *m*/*z* (%): 498 (M+2, 21), 496 (M^+^, 25). Anal. Calcd. for “C_23_H_21_ClN_6_O_3_S” (496): C, 55.59; H, 4.26; N, 16.91; Found: C, 55.59; H, 4.26; N, 16.91%.

*Ethyl-5-(1,5-dimethyl-3-oxo-2-phenyl-2,3-dihydro-1H-pyrazol-4-yl)methylene)hydrazono)-4-(4-nitrophenyl)-4,5-dihydro-1,3,4-thiadiazole-2-carboxylate* (**7**), yellowish brown crystals from dioxane (76%); m.p. 157–159 °C, FT-IR (KBr, cm^−1^): 1668 (N-C=O), 1725 (C=O), 1600 (C=N), 1560 (C=C); ^1^H-NMR (DMSO-d_6_): δ 1.30 (t, 3H, CH_2_CH_3_), 2.48 (s, 3H, CH_3_), 3.29 (s, 3H, N-CH_3_), 4.33 (d, 2H, CH_2_CH_3_), 7.33–7.57 (m, 9H, Ar-H), 8.35 (s, 1H, CH); ^13^C-NMR (100 MHz, DMSO-d_6_): δ 12.28 (CH_3_), 12.37 (CH_2_CH_3_), 13.95 (N-CH_3_), 61.97 (CH_2_CH_3_), 86.40 (Ar), 123.27 (Ar), 125.18(Ar), 127.77(Ar), 129.12(Ar), 129.16 (Ar), 133.18 (Ar), 134.28 (Ar), 138.22 (Ar), 149.15 (Ar), 151.96 (Ar), 157.33 (Ar), 163.32 (CH), 163.77 (C=O), 165.82 (C=O); MS *m*/*z* (%): 507(M^+^, 72). Anal. Calcd. for “C_23_H_21_N_7_O_5_S” (507): C, 54.43; H, 4.17; N, 19.32; Found: C, 54.47; H, 4.12; N, 19.28%.

*5-(1,5-Dimethyl-3-oxo-2-phenyl-2,3-dihydro-1H-pyrazol-4-yl)methylene)hydrazono)-*N*-phenyl-4-(*p*-tolyl)-4,5-dihydro-1,3,4-thiadiazole-2-carboxamide* (**8**), yellow crystals from ethanol (68%); m.p. 260–262 °C, FT-IR (KBr, cm^−1^): 3315(NH), 1662 (N-C = O), 1680 (C=O), 1615 (C=N), 1598 (C=C); ^1^H-NMR (DMSO-d_6_): δ 2.31(s, 3H, CH_3_), 2.45 (s, 3H, CH_3_), 3.32 (s, 3H, N-CH_3_), 7.31–8.06 (m, 14H, Ar-H), 8.15 (s, 1H, CH), 10.63 (s, 1H, NH); ^13^C-NMR (100 MHz, DMSO-d_6_): δ 12.36 (CH_3_), 20.51 (CH_3_), 34.06 (N-CH_3_), 100.12 (Ar), 120.89 (Ar), 121.72 (Ar), 124.50 (Ar), 125.62(Ar), 127.52(Ar), 128.58 (Ar), 129.12(Ar), 129.20(Ar), 146.61(Ar), 147.00 (Ar), 148.63 (Ar), 156.24 (C=O), 162.24 (CH), 163.45(C=O); MS *m*/*z* (%): 507(M^+^, 72). Anal. Calcd. for “C_28_H_25_N_7_O_2_S” (523): C, 64.23; H, 4.81; N, 18.73; Found: C, 64.19; H, 4.78; N, 18.68 %.

*5-(1,5-Dimethyl-3-oxo-2-phenyl-2,3-dihydro-1H-pyrazol-4-yl)methylene)hydrazono)-4-(4-nitrophenyl)-*N*-phenyl-4,5-dihydro-1,3,4-thiadiazole-2-carboxamide* (**9**), brown crystals from ethanol (63%); m.p. 281–283 °C, FT-IR (KBr, cm^−1^): 3322(NH), 1668 (N-C=O), 1680(C=O), 1615 (C=N), 1600 (C=C); ^1^H-NMR (DMSO-d_6_): δ 2.50 (s, 3H, CH_3_), 3.28 (s, 3H, N-CH_3_), 7.21–7.78 (m, 14H, Ar-H), 8.08 (s, 1H, CH), 10.79 (s, 1H, NH); ^13^C-NMR (100 MHz, DMSO-d_6_): δ 12.36 (CH_3_), 32.12 (N-CH_3_), 98.14 (Ar), 121.89 (Ar), 122.74 (Ar), 123.51 (Ar), 124.52 (Ar), 126.82 (Ar), 127.61(Ar), 128.15 (Ar), 128.28 (Ar), 145.62 (Ar), 146.50 (Ar), 149.83 (Ar), 155.24(C=O), 163.24 (CH), 163.55 (C=O); MS *m*/*z* (%): 554 (M^+^, 11). Anal. Calcd. for “C_27_H_22_N_8_O_4_S” (554): C, 58.48; H, 4.00; N, 20.21; Found: C, 58.42; H, 3.95; N, 20.17%.

*Ethyl 4-(1,5-dimethyl-3-oxo-2-phenyl-2,3-dihydro-1H-pyrazol-4-yl)-6-methyl-2-thioxo-1,2,3,4-tetrahydropyrimidine-5-carboxylate* (**10**): A mixture of 1,5-dimethyl-3-oxo-2-phenyl-2,3-dihydro-1*H*-pyrazole-4-carbaldehyde (**1**) (1.08 g, 5 mmol), ethyl acetoacetate (0.65 mL, 5 mmol), and thiourea (0.35 g, 5 mmol) was dissolved in 15 mL DMF with the addition of a few drops of sulfuric acid. The reaction mixture was heated under reflux for 5hrs. After cooling, the solid formed was separated and recrystallized from dioxane as yellow crystals; Yield (83%); m.p. 212–214 °C, FT-IR (KBr, cm^−1^): 3415, 33821(NH), 668 (N-C=O), 1735(C=O), 1620 (C=N), 1600 (C=C); ^1^H-NMR (DMSO-d*_6_*): *δ* 1.02(t, 3H, CH_2_CH_3_), 2.17(s, 6H, 2CH_3_), 3.23 (s, 3H, N-CH_3_), 3.50(s, 1H, CH-4, pyrimidine), 3.96(q, 2H, CH_2_CH_3_), 7.29–7.45 (m, 5H, Ar-H), 12.23 (s, 1H, NH), 12.29 (s, 1H, NH); ^13^C-NMR (100 MHz, DMSO-d*_6_*): *δ* 13.57 (CH_2_CH_3_), 15.58 (CH_3_), 18.54 (CH_3_), 52.02 (CH-4, pyrimidine), 61.66 (CH_2_CH_3_), 103.84 (Ar), 106.44 (Ar), 120.20(Ar), 124.41(Ar), 126.92(Ar), 134.47(Ar), 153.09 (Ar), 160.98 (Ar), 166.18(C=O), 167.89(C=O), 176.14 (C=S); MS *m*/*z* (%): 386 (M^+^, 71). Anal. Calcd. for “C_19_H_22_N_4_O_3_S” (386): C, 59.05; H, 5.74; N, 14.50; Found: C, 58.98; H, 5.72; N, 14.44%.

*4-(1,5-dimethyl-3-oxo-2-phenyl-2,3-dihydro-1H-pyrazol-4-yl)-6-methyl-2-thioxo-1,2,3,4-tetrahydropyrimidine-5-carbohydrazide* (**11**): Ethyl 4-(1,5-dimethyl-3-oxo-2-phenyl-2,3-dihydro-1H-pyrazol-4-yl)-6-methyl-2-thioxo-1,2,3,4-tetrahydropyrimidine-5-carboxylate (**10**) (1.9 g, 5 mmol) and hydrazine hydrate (5mmol) were mixed in 20 mL absolute ethanol. The reaction mixture was heated under reflux for 5hrs. After cooling, the solid formed was separated and recrystallized from ethanol as white crystals; Yield (85%); m.p. 225–227 °C, FT-IR (KBr, cm^−1^): 3415 (NH), 3386 (broad, NH, NH_2_),1664 (N-C=O), 1682 (C=O), 1620 (C=N), 1590 (C=C); ^1^H-NMR (DMSO-d*_6_*): *δ* 2.12 (s, 6H, 2CH_3_), 3.34 (s, 3H, CH_3_), 3.65 (s, 1H, CH-4, pyrimidine), 5.36 (s, 2H, NH_2_), 6.15–6.55 (m, 5H, Ar-H), 8.06 (s, 1H, NH), 8.79 (s, broad, 2H, 2NH); ^13^C-NMR (100 MHz, DMSO-d*_6_*): *δ* 15.72 (CH_3_), 17.94(CH_3_), 53.22 (CH-4, pyrimidine), 104.84 (Ar), 108.44 (Ar), 121.22 (Ar), 123.61(Ar), 124.92(Ar), 133.57(Ar), 143.09(Ar), 152.98(Ar), 164.58(C=O), 165.19 (C=O), 182.12 (C=S); MS *m*/*z* (%): 372(M^+^, 21). Anal. Calcd. for “C_17_H_20_N_6_O_2_S” (372): C, 54.82; H, 5.41; N, 22.56; Found: C, 54.86; H, 5.37; N, 22.51%.

*1,5-dimethyl-4-(6-methyl-5-(3-methyl-5-oxo-4,5-dihydro-1H-pyrazole-1-carbonyl)-2-thioxo-1,2,3,4-tetrahydropyrimidin-4-yl)-2-phenyl-1,2-dihydro-3H-pyrazol-3-one* (**12**): 4-(1,5-dimethyl-3-oxo-2-phenyl-2,3-dihydro-1*H*-pyrazol-4-yl)-6-methyl-2-thioxo-1,2,3,4-tetra hydropyrimidine-5-carbohydrazide (**11**) (1.86 g, 5 mmol) and ethyl acetoacetate (0.65 mL, 5 mmoL) were refluxed in 20 mL ethanol containing 1–3 drops of acetic acid for 5h; the solid formed while heating was separated and recrystallized from acetic acid as beige crystals. Yield: 71%, m.p. 271–273 °C, FT-IR (KBr, cm^−1^): 3412, 3374 (NH), 1680 (broad, C=O), 1665 (N-C = O), 1600 (C=N), 1560 (C=C); ^1^H-NMR (DMSO-d*_6_*): *δ* 1.94 (s, 3H, CH_3_), 2.26 (s, 6H, 2CH_3_), 3.12 (d, 2H, CH_2_-pyrazole), 3.34 (s, 3H, CH_3_), 3.95(s, 1H, CH-4, pyrimidine), 7.32–7.52 (m, 5H, Ar-H), 8.25 (s, 1H, NH), 10.01 (s, 1H, NH); ^13^C-NMR (100 MHz, DMSO-d*_6_*): *δ* 15.72 (CH_3_), 17.94 (CH_3_), 42.15 (CH_2_-pyrazole), 51.22 (CH-4, pyrimidine), 106.14 (Ar), 108.24 (Ar), 120.12 (Ar), 121.61 (Ar), 122.12 (Ar), 123.01 (Ar), 134.57 (Ar), 143.09 (Ar), 152.98 (Ar), 159.14 (Ar), 163.24 (C=O), 164.58 (C=O), 165.19 (C=O), 182.12 (C=S); MS *m*/*z* (%): 438 (M^+^, 51). Anal. Calcd. for “C_21_H_22_N_6_O_3_S” (438): C, 57.52; H, 5.06; N, 19.17; Found: C, 57.56; H, 5.02; N, 19.12%.

### 3.2. Antimicrobial Activity

#### 3.2.1. Antimicrobial Assay

The agar well diffusion method was used to test the antimicrobial activity of the synthesized samples against six microorganisms, including Gram-negative bacteria (*Escherichia coli* ATCC 25922 and *Pseudomonas aeruginosa* ATCC 27853), Gram-positive bacteria (*Staphylococcus aureus* ATCC 25923 and *Bacillus subtilis* ATCC 6051), and unicellular fungi (*Candida albicans* ATCC 90028 and *Cryptococcus neoformans* ATCC 14116). 

The pathogenic microorganisms that were screened were known to cause UTIs. While the fungal strains were seeded on malt extract agar (MEA) plates and cultured for 3–5 days at 28 ± 2 °C before being maintained at 4 °C for subsequent usage, the examined bacteria were inoculated on nutrient agar for one day at 37 °C [49]. The minimum inhibitory concentration (MIC) of the synthesized samples was also established using the microdilution assay [50]. Briefly, the tested microbes were grown on nutrient agar, and three or four colonies were suspended in fresh sterile nutrient broth to achieve a concentration of 1 × 10^8^ CFU/mL. Each well of a 96-well microtiter plate was filled with one hundred microliters of the 1:100 diluted cell suspensions. The MIC was calculated using various doses for each sample (from 1000 µg/mL to 0.5 µg/mL).

The specific and fixed wavelength used for detecting the optical density for the bacterial growth (at 600 nm) was completely different from the optical density for the synthesized sample. A negative control (the nutrient broth alone) and positive control (the tested microbes and the nutrient broth) were conducted for these purposes; additionally, another control like the synthesized sample alone was used to compare the conducted results as an auto-zero purpose. The MICs were determined as the lowest sample dilution capable of inhibiting visible bacterial growth.

To evaluate the antimicrobial capability of the synthesized materials, the ZOI test must be combined with the use of nystatin (NS) as an antifungal positive control and amoxicillin/clavulanic acid (7:1 ratio; AMC), a common antibacterial drug.

#### 3.2.2. Growth Curve Assay 

According to Huang et al., the growth curve test was used to investigate how chemical 9 affected the growth of *E. coli*, the most sensitive bacteria. In 5.0 mL nutrient broth tubes, the bacterial suspension was adjusted to 0.5 McFarland (1 × 10^8^ CFU/mL). Each tube under inspection had a single addition of compound 9. At 2 h intervals up to 24 h after treatment, the absorbance of the bacterial growth was measured (wavelength: 600 nm). To obtain the typical growth curve, the average of the triplicate data was compared to the hourly intervals [51,52].

#### 3.2.3. Effect of Compound 9 on Protein Leakage from Bacterial Cell Membranes

Furthermore, 0.5 McFarland (1 × 10^8^ CFU/mL) was chosen for the pure 18 h bacterial culture, and 100 µL of it was put into 10 mL of the nutritional broth containing compound 9. As a control, a broth devoid of component 9 was infused with culture. All of the treated samples were centrifuged for 15 min at 5000× *g* rpm after being incubated at 37 °C for 5 h. A total of 100 µL of supernatants and 1 mL of Bradford reagent were blended for each of the various samples. At 595 nm, optical density was measured following 10 min of incubation in the dark. 

#### 3.2.4. Mechanism of Biological Action Using SEM Analysis 

The bacterial suspensions were adjusted to 0.5 McFarland (1 × 10^8^ CFU/mL) in nutrient broth tubes, and about 5.0 mL of the adjusted bacterial suspension was mixed with 1.0 mL of the prepared compound 9 at a concentration of 15.62 µg/mL (MIC result in Table 1) and incubated at 37 ± 2 °C for about 24 h. It must be noted that a non-treated bacterial suspension was prepared as previously mentioned without the addition of compound 9. All of the treated and non-treated samples were centrifuged for 15 min at 5000× *g* rpm, and the cell-free supernatants were discarded and the bacterial cells were collected. After three PBS washes of the collected cells, the delicate *E. coli* was preserved in a 4.0% glutaraldehyde solution. The stored microbial cells were periodically drained with ethanol concentrations of 30, 50, 70, 90, and 100% for 15 min at 28 ± 2 °C, followed by PBS cleaning. The fixed samples were then cemented on a piece of aluminum for SEM examination. SEM analysis was used to determine the morphological shape of the control (non-treated microbial cell) and treated microorganisms.

#### 3.2.5. Statistical Analysis 

The statistical analysis of the obtained results was investigated after applying a one-way ANOVA (at *p* < 0.05) and Duncan’s methods. SPSS software version 15 was used to examine the accepted results. It must be noted that all the biological tests were repeated three times.

### 3.3. In Silico Studies 

The 2D structures of compounds were sketched using ChemDraw 16 and then converted to 3D structures and minimized using OpenBabel GUI [53]. The target enzyme was downloaded from the protein data bank (https://www.rcsb.org/) [54] under PDB code 1IP6 for *E. coli* β-carbonic anhydrase [55]. The file was energy-minimized using the CHARMM Force Field [56] in Discovery Studio. A molecular docking study was carried out through the PyRx- Virtual screening tool [57]. The dug-likeness properties of targeted compounds were calculated using SwissADME and admetSAR tools. 

## 4. Conclusions

The present study overlays the synthesis of a new series of thiadiazole and pyrimidine moieties attached to antipyrine analogue 2–12, and their structures were confirmed via spectral and elemental analyses. The antimicrobial potential was estimated through in vitro ZOI and MIC. On the other hand, the antibacterial reaction mechanism was tested through a membrane leakage assay and SEM imaging technique. In comparison to AMC/Nyst, the industry standard antimicrobial agent, all developed compounds showed promising antibacterial activity against all of the evaluated bacterial and fungal species. In comparison to AMC/Nyst, compounds 4, 5, and 9 were noticeably more active. With inhibition zones of 19.0, 14.2, 17.0, 15.0, 16.9, and 9.5 mm, respectively, compound 9 at a dosage of 0.1 mg/mL showed potential antibacterial action against *E. coli*, *P. aeruginosa*, *S. aureus*, *B. subtilis*, *C. albicans*, and *C. neoformans*, whereas the MICs for compounds 9, 5, and 4 were 15.62, 31.25, and 125 µg/mL, respectively. It was discovered that the amount of cellular protein released from *E. coli* is directly correlated to the quantity of compound 9, which was found to be 177.99 µg/mL following treatment with 1.0 mg/mL of compound 9. This finding supports compound 9’s antibacterial properties and explains how the formation of pits in the *E. coli* cell membrane results in the release of proteins from the cytoplasm. A substance must stick to its target site on microbial cells and establish a specific number of crucial regions associated with its level within pathogenic microorganisms in order for it to possess antimicrobial potentials that may eliminate those pathogenic microbes. The docking studies proved that compound 9 declared a greater activity than other synthesized compounds.

## Figures and Tables

**Table 1:** Antimicrobial action and MIC of the manufactured compounds upon various pathogenic bacteria and yeasts.

Test Organism	Compound 4	Compound 5	Compound 9			AMC/Nystatin		
	IZ (mm)	MIC µg/mL	IZ (mm)	MIC µg/mL	IZ (mm)	MIC µg/mL	IZ (mm)	MIC µg/mL
*E. coli*	14.0 ± 0.55 ^e^	125	17.5 ± 0.76 ^e^	31.25	19.0 ± 0.58 ^f^	15.62	15.0 ± 0.46 ^d^	250
*P. aeruginosa*	9.5 ± 0.40 ^b^	250	11.2 ± 0.58 ^d^	125	14.2 ± 1.53 ^b,c,d^	125	20.0 ± 1.00 ^c^	500
*S. aureus*	13.1 ± 0.85 ^d^	125	11.3 ± 0.53 ^d^	125	17.0 ± 0.76 ^d,e^	31.25	28.3 ± 0.50 ^b^	250
*B. subtilis*	8.4 ± 0.98 ^b,c^	500	9.6 ± 0.87 ^b,c^	500	15.0 ± 0.50 ^a^	31.25	27.0 ± 1.00 ^a^	31.25
*C. albicans*	10.2 ± 0.72 ^f^	250	11.0 ± 0.45 ^f^	125	16.9.0 ± 0.58 ^f^	31.25	10.3 ± 0.58 ^f^	125
*C. neoformans*	8.5 ± 0.68 ^b,c,d^	500	8.8 ± 0.69 ^c^	500	9.5 ± 1.15 ^c,d,e^	500	18.8 ± 0.68 ^c^	250

IZ: inhibition zone, MIC: minimum inhibition concentration, AMC: amoxicillin/clavulanic acid. Values are means ± standard error (*n* = 3). Data within the groups are analyzed using one-way analysis of variance (ANOVA) followed by ^a,b,c,d,e,f^ Duncan’s multiple range test (DMRT).

**Table 2:** Binding energies of the compounds against *E. coli* β-carbonic anhydrase.

Entry	Binding Energy (kcal/mol)	Docked Complex (Amino Acid–Ligand) Interactions	Distance (Å)
**1**	−7.1	H-bonds	
		ASN46:ND2—compound **1**	2.99
		ASN60:ND2—compound **1**	3.07
		Arene–arene	
		TRP109—compound **1**	3.93
		TRP64—compound **1**	4.94
**3**	−7.0	Arene–arene	
		TRP109—compound **3**	3.84
		TRP64—compound **3**	4.64
**4**	−8.1	H-bonds	
		VAL110:N—compound **4**	2.80
		ALA111:N—compound **4**	2.85
**5**	−9.2	H-bonds	
		TRP64:NE1—compound **5**	2.81
		Arene–arene	
		TYR63—compound **5**	5.71
**6**	−8.1	H-bonds	
		GLN104:NE2—compound **6**	2.94
		ARG113:NH2—compound **6**	2.99
		Arene–arene	
		TRP109—compound **6**	4.80
		TRP64—compound **6**	5.82
		TYR63—compound **6**	3.84
**7**	−8.8	H-bonds	
		TRP64:NE1—compound **7**	2.86
		Arene–arene	
		TYR63—compound **7**	5.60
**8**	−9.3	H-bonds	
		TRP64:NE1—compound **8**	2.44
		GLU35:OE1—compound **8**	2.49
		Arene–arene	
		TYR63—compound **8**	4.18
		Arene–sigma	
		TYR63—compound **8**	3.50
**9**	−9.7	H-bonds	
		TRP64:NE1—compound **9**	2.91
		Arene–sigma	
		TYR63—compound **9**	3.70
**10**	−7.7	H-bonds	
		TRP64:NE1—compound **10**	2.79
**11**	−7.5	H-bonds	
		TRP64:NE1—compound **11**	3.00
**12**	−8.8	H-bonds	
		TRP64:ND2—compound **12**	3.12
		TRP64:ND2—compound **12**	3.01
		SER51:OG—compound **12**	2.78
		Arene–arene	
		TRP109—compound **12**	3.85
		TRP64—compound **12**	4.64
**Ciprofloxacin**	−7.6	H-bonds	
		TRP64:NE1—ciprofloxacin	3.10
		TRP64:NE1—ciprofloxacin	3.19
		TRP109:NE1—ciprofloxacin	2.78

**Table 3:** The physicochemical and pharmacokinetics properties of the compounds.

	Molecular Weight (g/mol)	BBB Permeant	GI Absorption	%Human Intestinal Absorption (HIA+)	logp	TPSA A^2^	HBA	HBD	N Rotatable	N Violations	Bioavailability Score	AMES Toxicity	Carcinogenicity
Ref. Range	130–500			<25 poor >80 high	≤5	≤140	2.0–20.0	0.0–6.0	≤10	≤1		Nontoxic	Noncarcinogenic
**1**	216.24	Yes	high	98.26	1.12	44.00	2	0	2	0	0.55	Nontoxic	Noncarcinogenic
**3**	336.48	No	high	94.12	2.69	108.71	2	1	5	0	0.55	Nontoxic	Noncarcinogenic
**4**	432.50	No	high	98.59	2.89	114.78	5	0	5	0	0.55	Nontoxic	Noncarcinogenic
**5**	477.50	No	low	92.77	2.06	160.60	7	0	6	0	0.55	Nontoxic	Noncarcinogenic
**6**	496.97	No	high	98.42	3.88	124.01	7	0	6	0	0.55	Nontoxic	Noncarcinogenic
**7**	507.52	No	low	89.60	2.60	169.83	8	0	8	1	0.17	Nontoxic	Noncarcinogenic
**8**	523.61	No	low	98.53	4.09	126.81	5	1	7	1	0.55	Nontoxic	Noncarcinogenic
**9**	554.58	No	low	93.03	3.09	172.63	7	1	8	1	0.17	Nontoxic	Noncarcinogenic
**10**	386.47	No	high	95.42	1.91	109.38	3	2	5	0	0.55	Nontoxic	Noncarcinogenic
**11**	372.44	No	low	88.71	1.05	138.20	3	4	4	0	0.55	Nontoxic	Noncarcinogenic
**12**	438.50	No	high	92.90	1.46	132.80	4	2	4	0	0.55	Nontoxic	Noncarcinogenic

## Data Availability

All available data is listed in this article and Supplementary Materials.

## Supplementary Materials

The following supporting information can be downloaded at: https://www.mdpi.com/article/10.3390/molecules28227491/s1, Figure S1–S18: H1-NMR and C13-NMR spectra of compounds **3**–**12**.
