# Supplementary material for: Antimicrobial Potency and E. coli β-Carbonic Anhydrase Inhibition Efficacy of Phenazone-Based Molecules
Source: Molecules. 2023 Nov 8;28(22):7491. doi: 10.3390/molecules28227491 (PMC10672871; doi:10.3390/molecules28227491)
Supplement: Supplementary file 1 [file molecules-28-07491-s001.zip › molecules-2566546-supplementary.pdf]

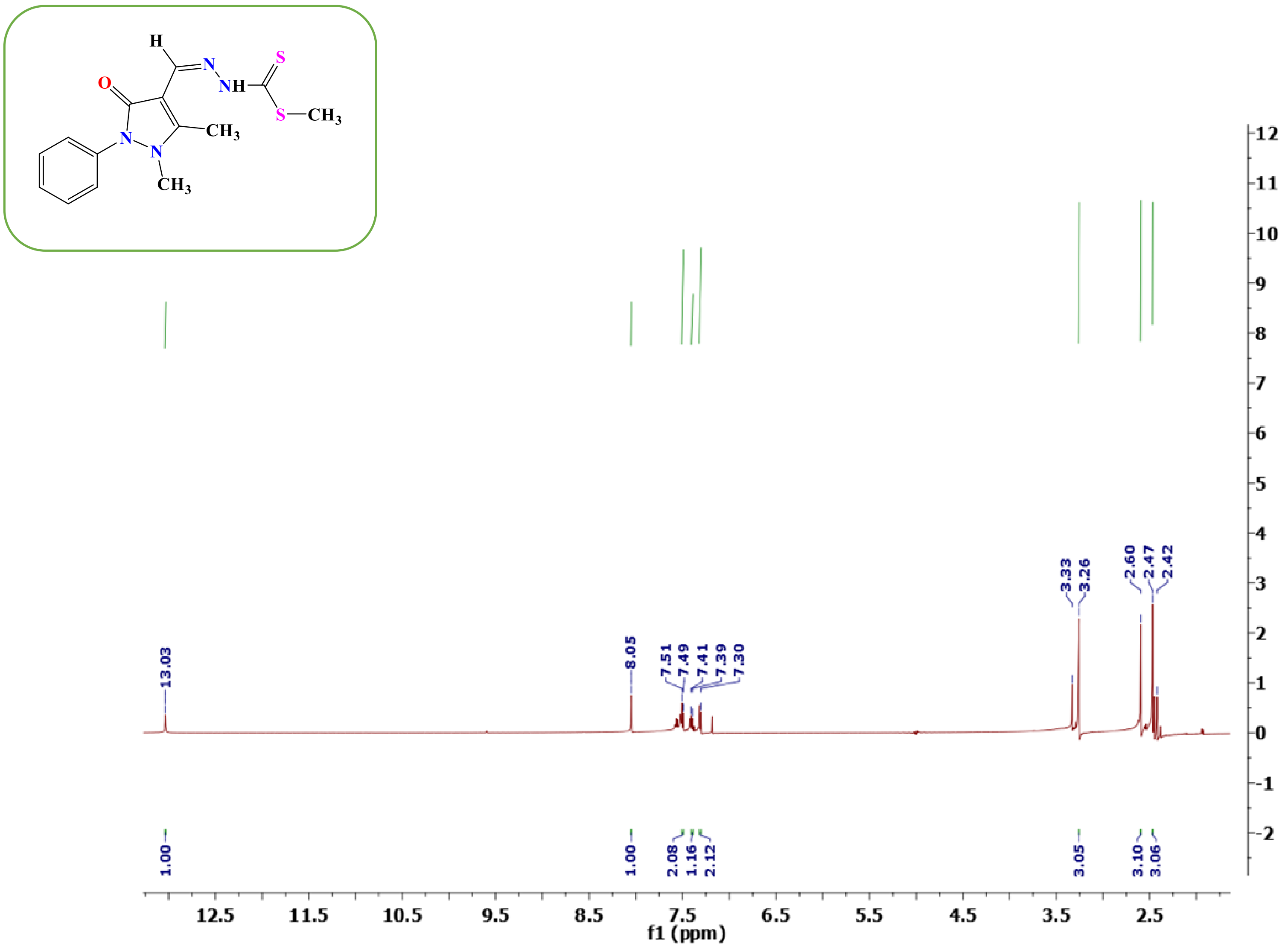

Figure S1: H[1]-NMR spectrum of compound no.3

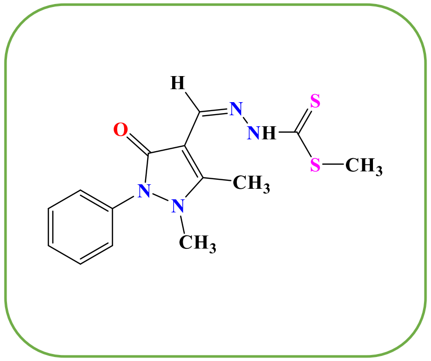

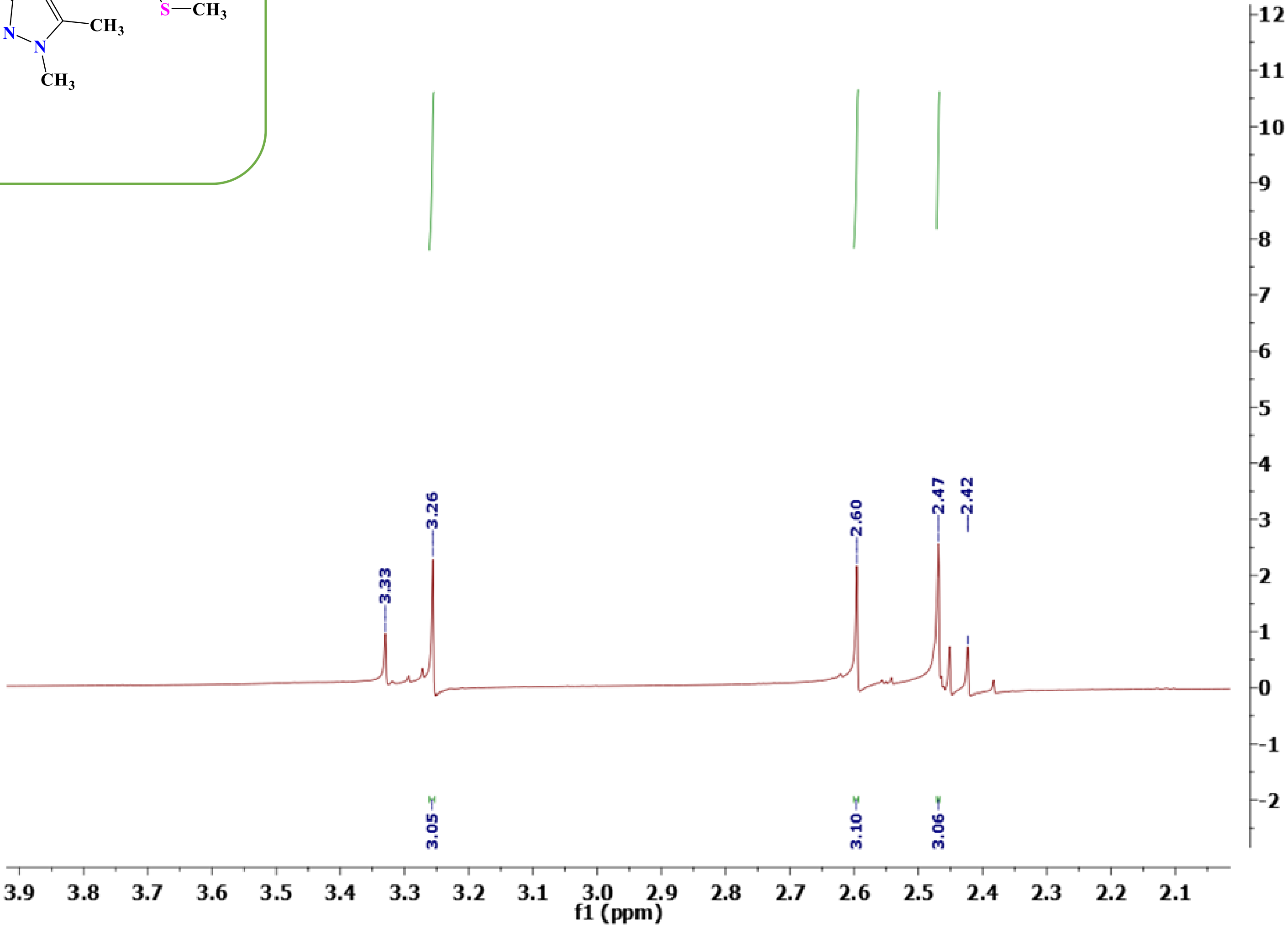

Figure S2: H[1]-NMR spectrum of compound no.3

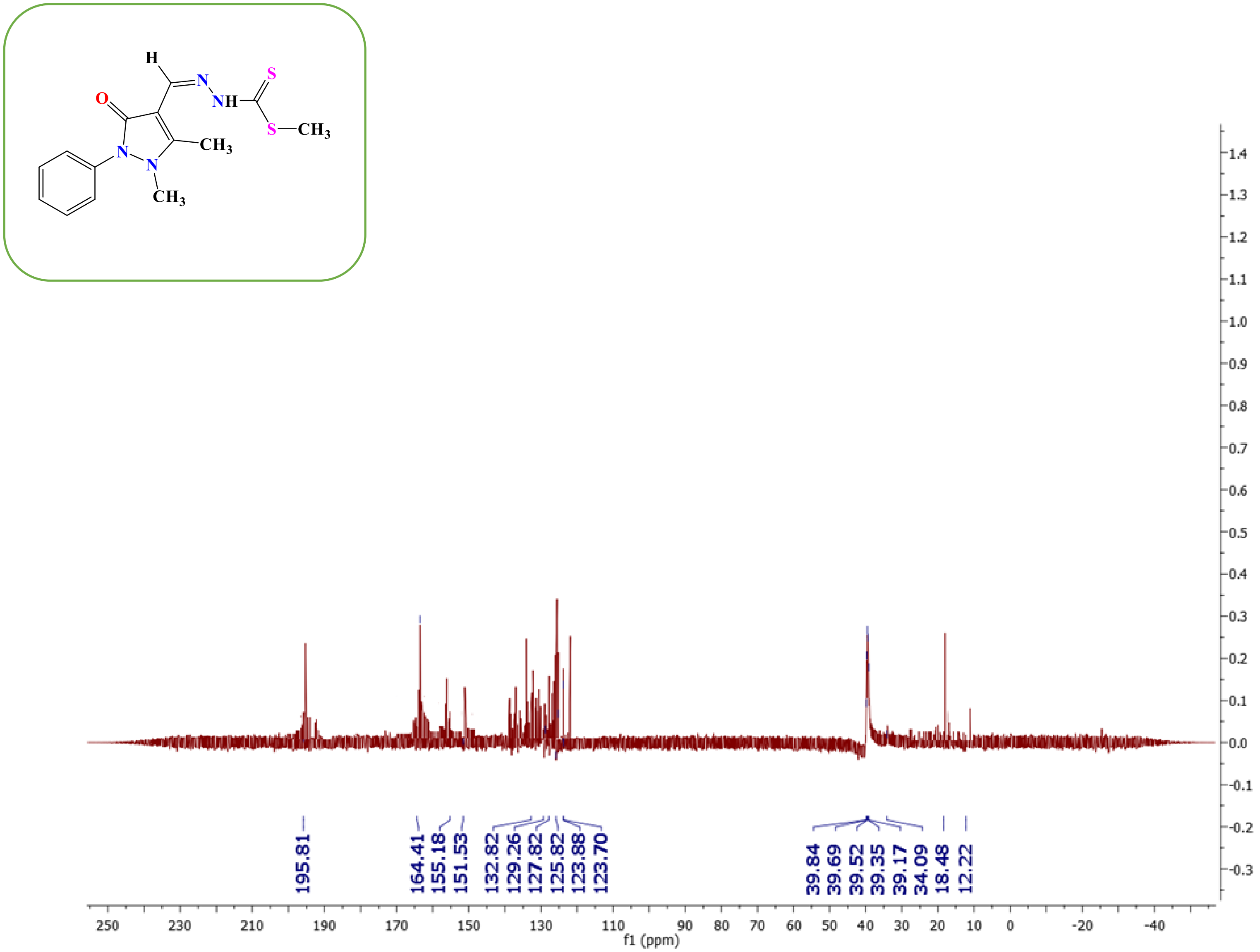

Figure S3: C13-NMR spectrum of compound no.3

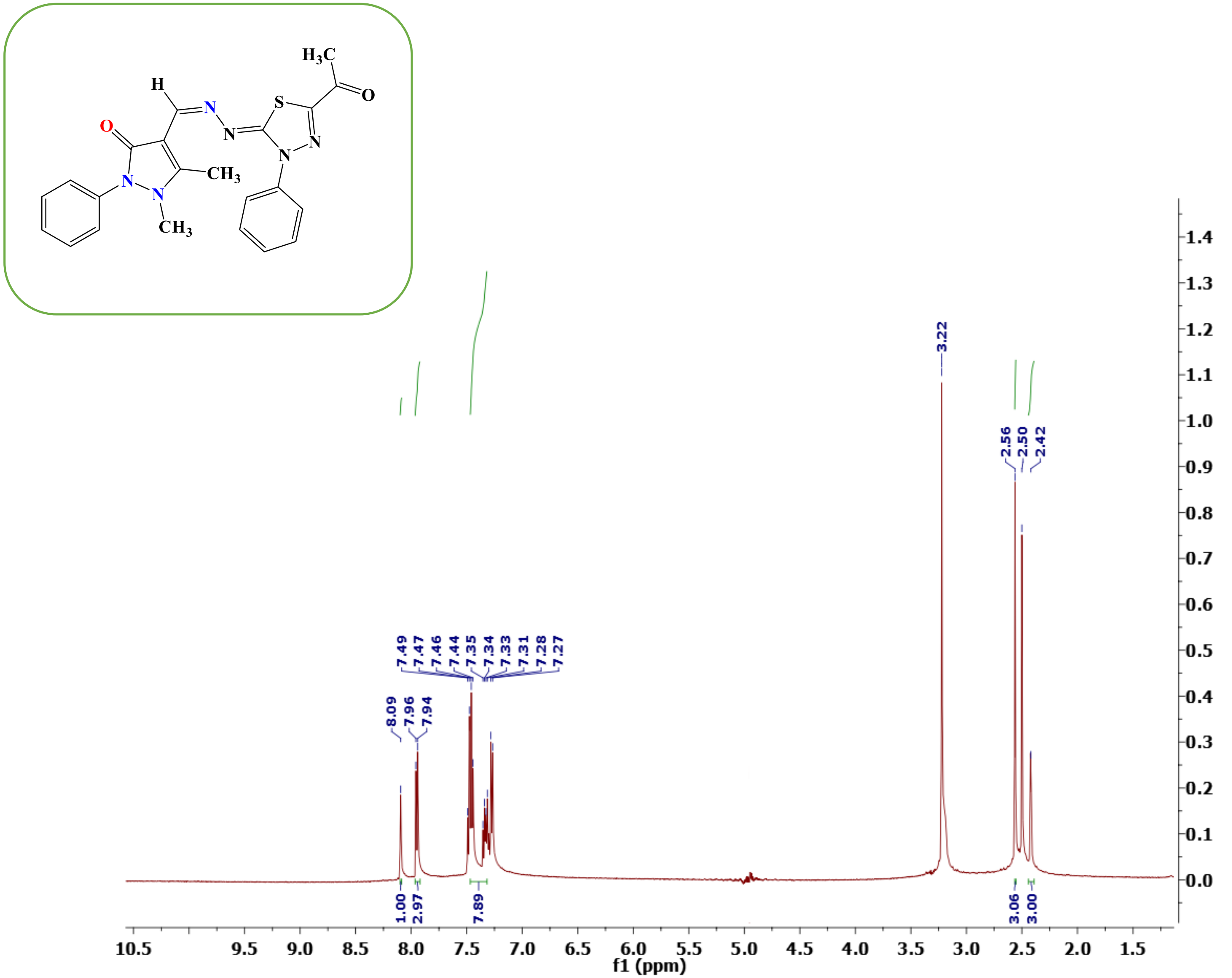

Figure S4: H1-NMR spectrum of compound no.4

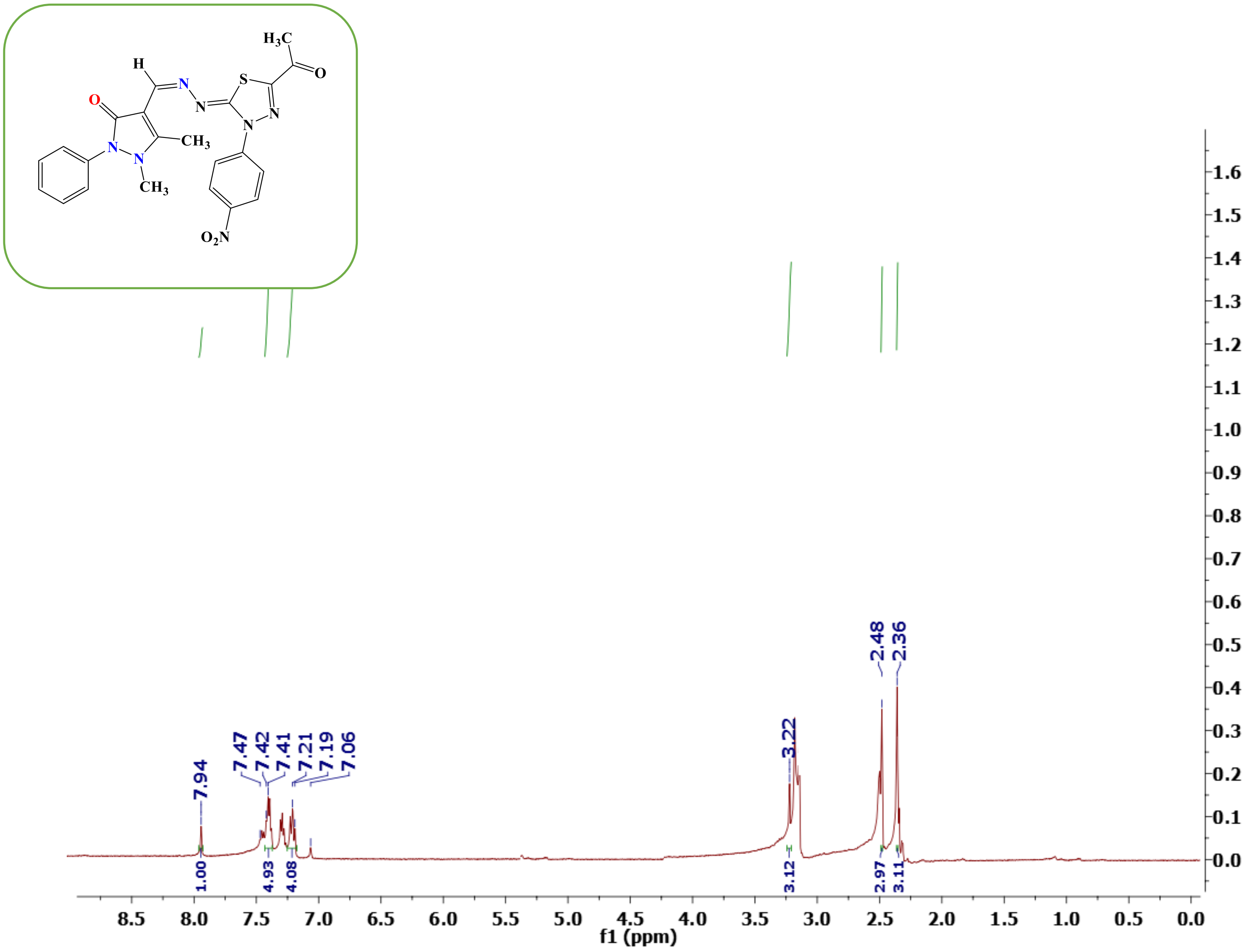

Figure S5: H1-NMR spectrum of compound no.5

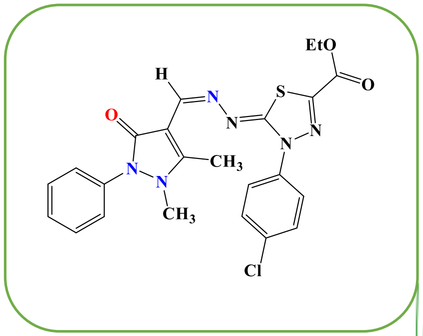

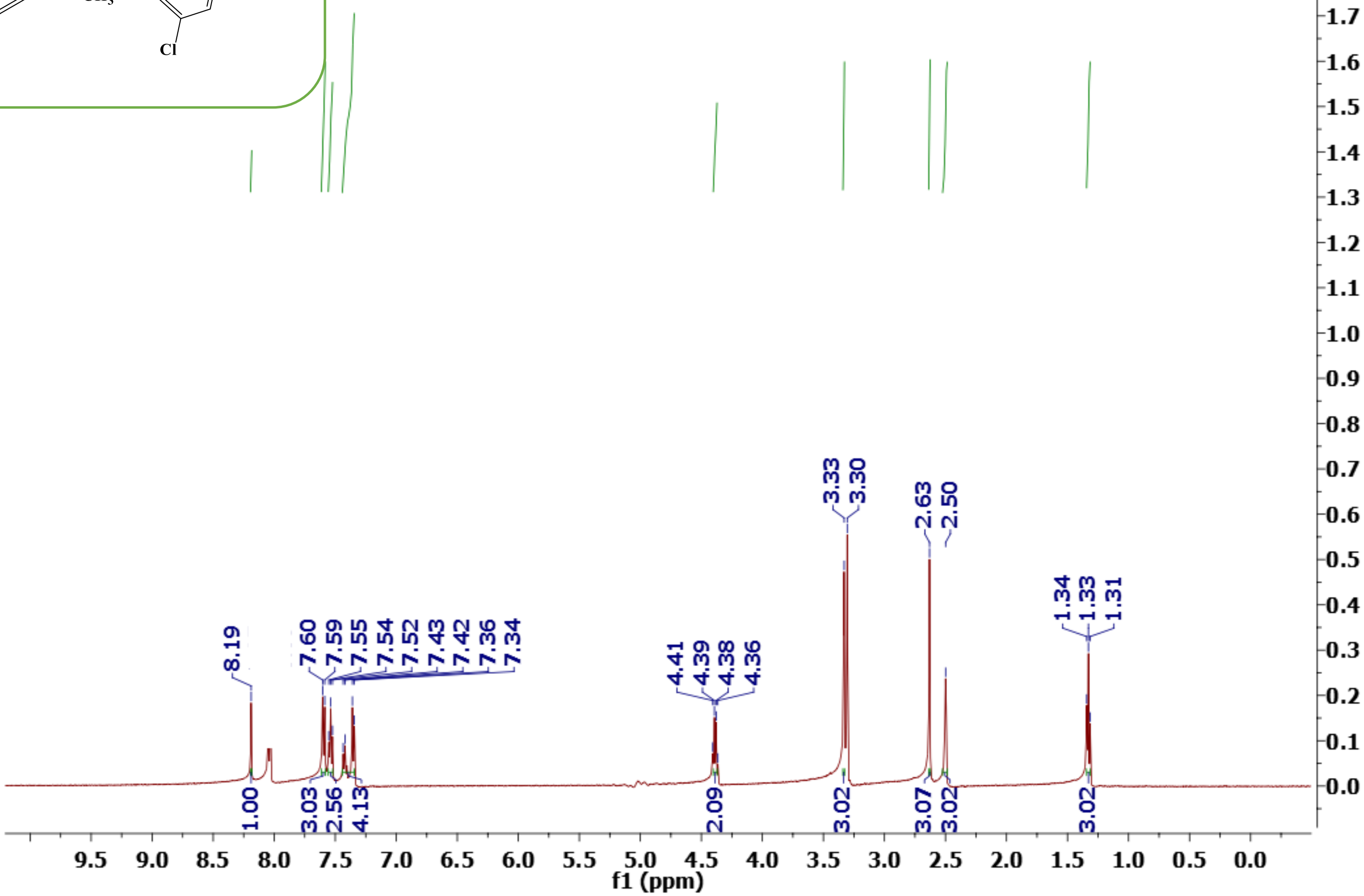

Figure S6: H1-NMR spectrum of compound no.6

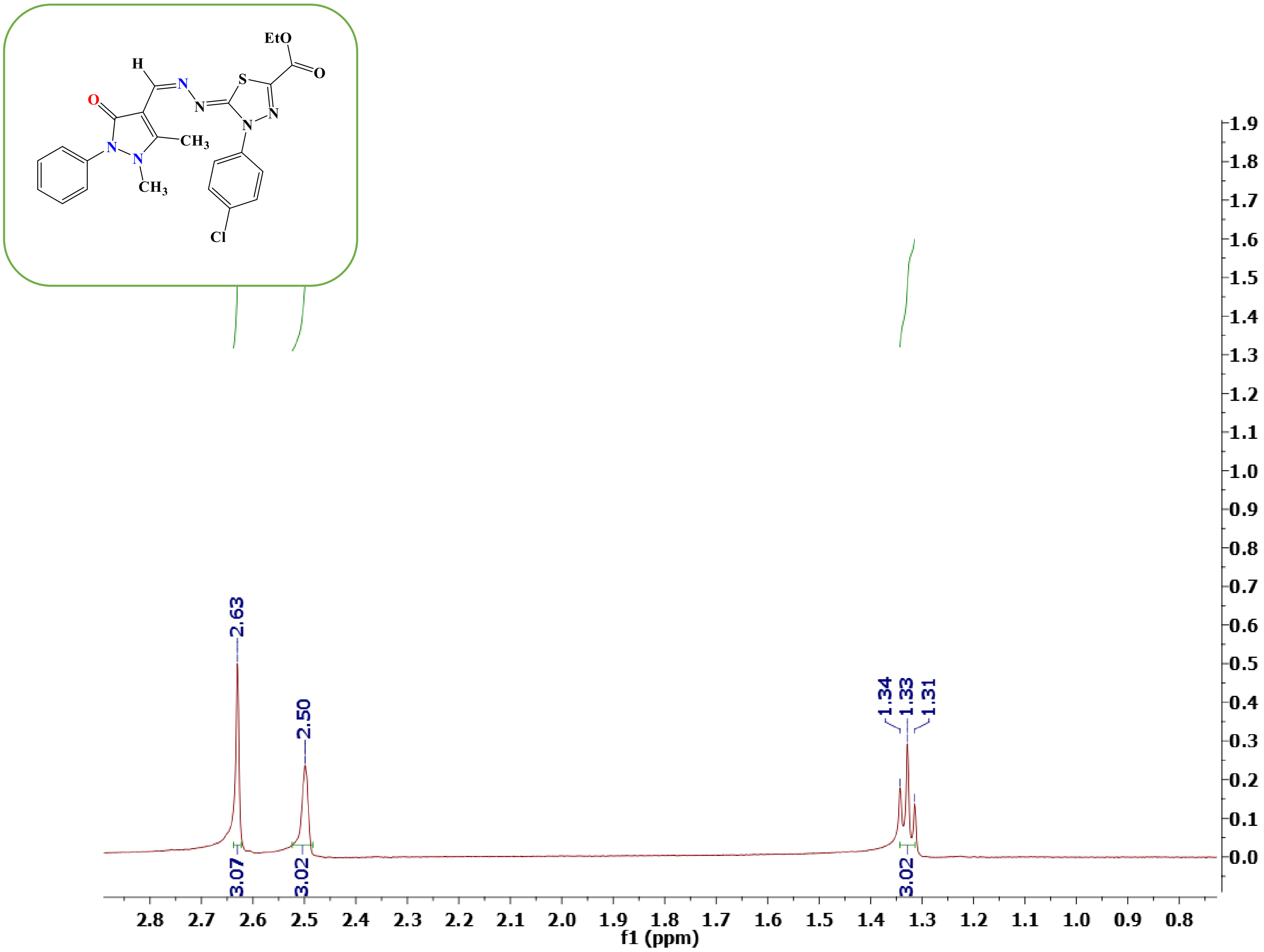

Figure S7: H1-NMR spectrum of compound no.6

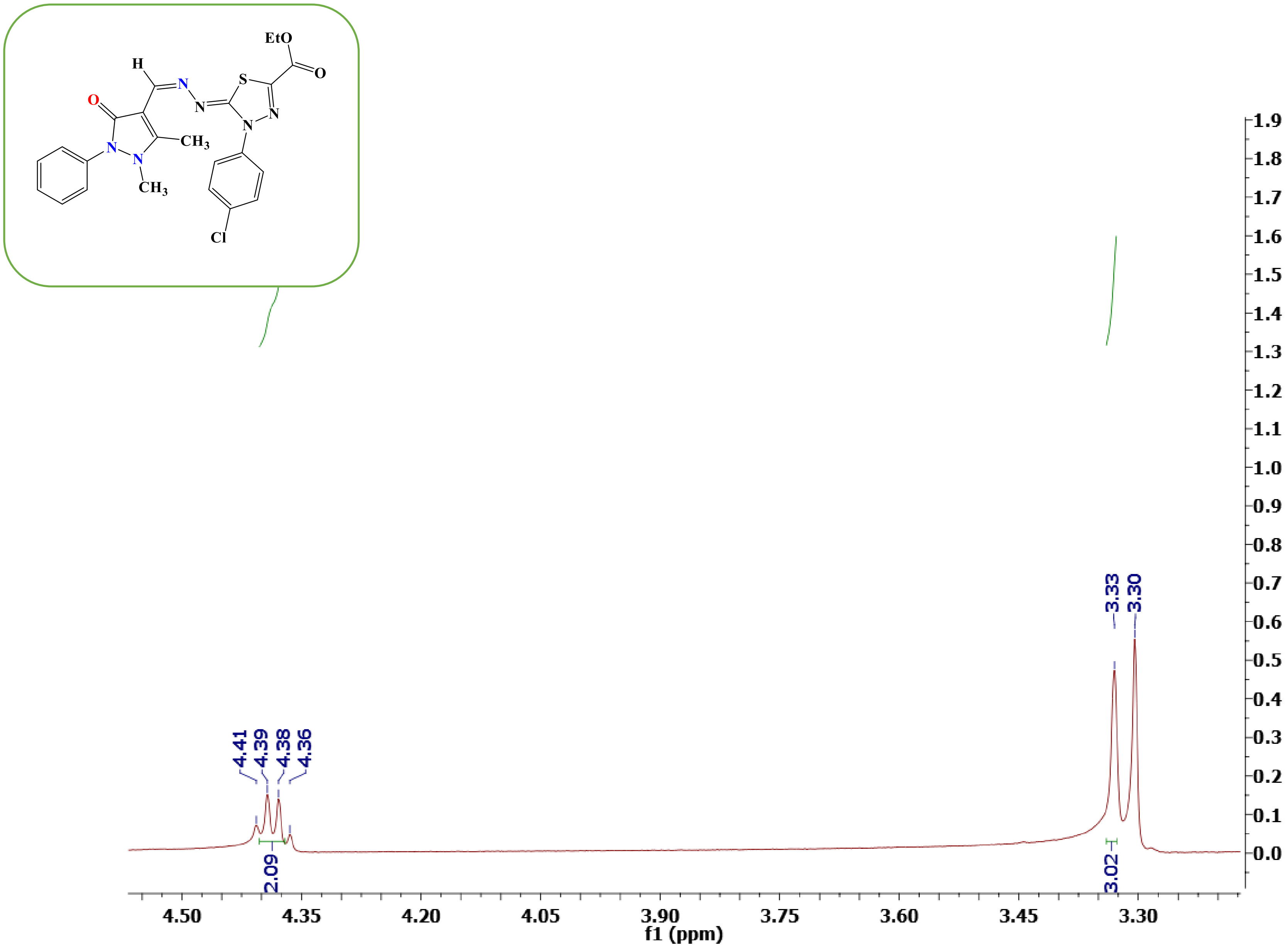

Figure S8: H1-NMR spectrum of compound no.6

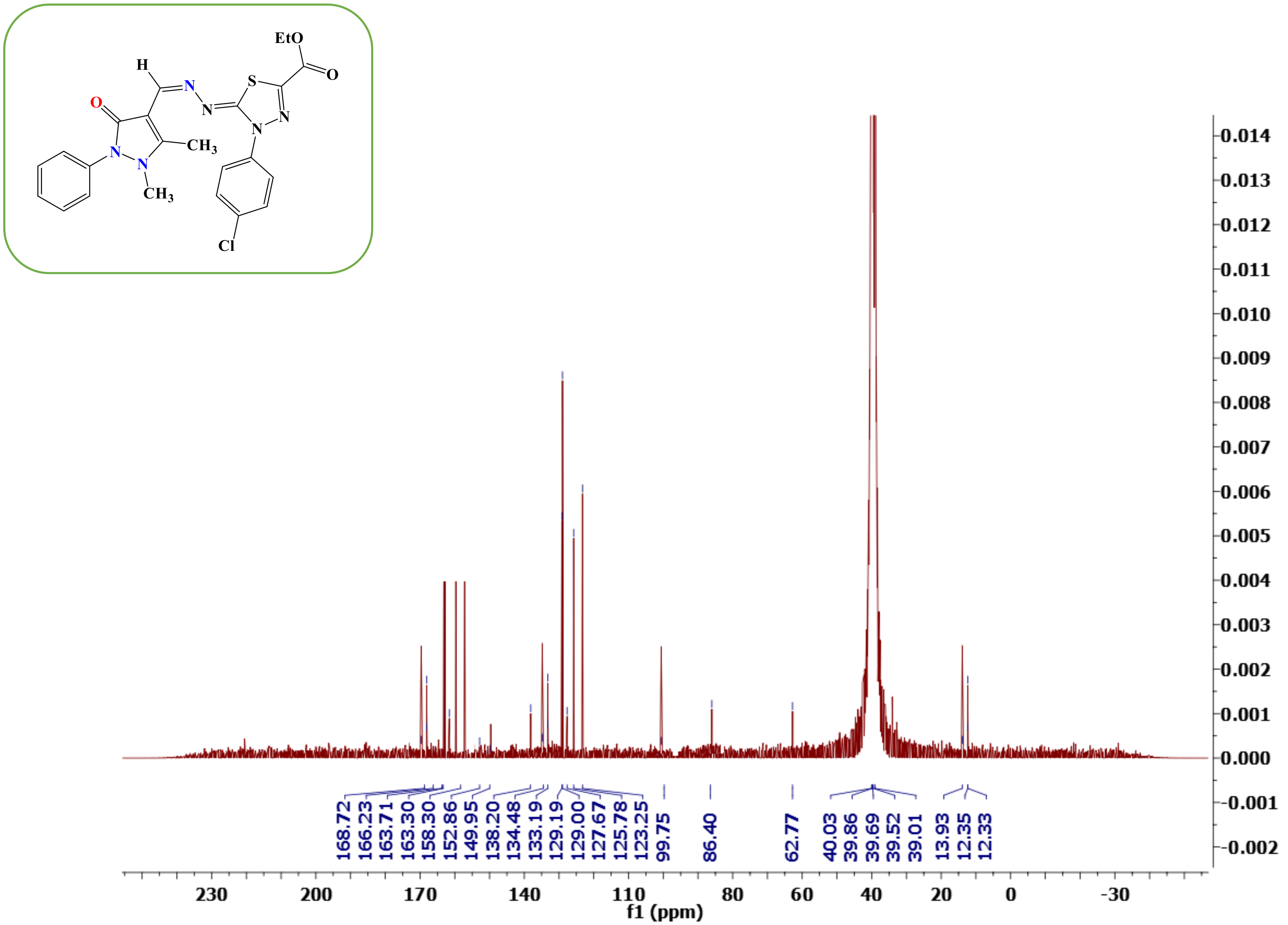

Figure S9: C13-NMR spectrum of compound no.6

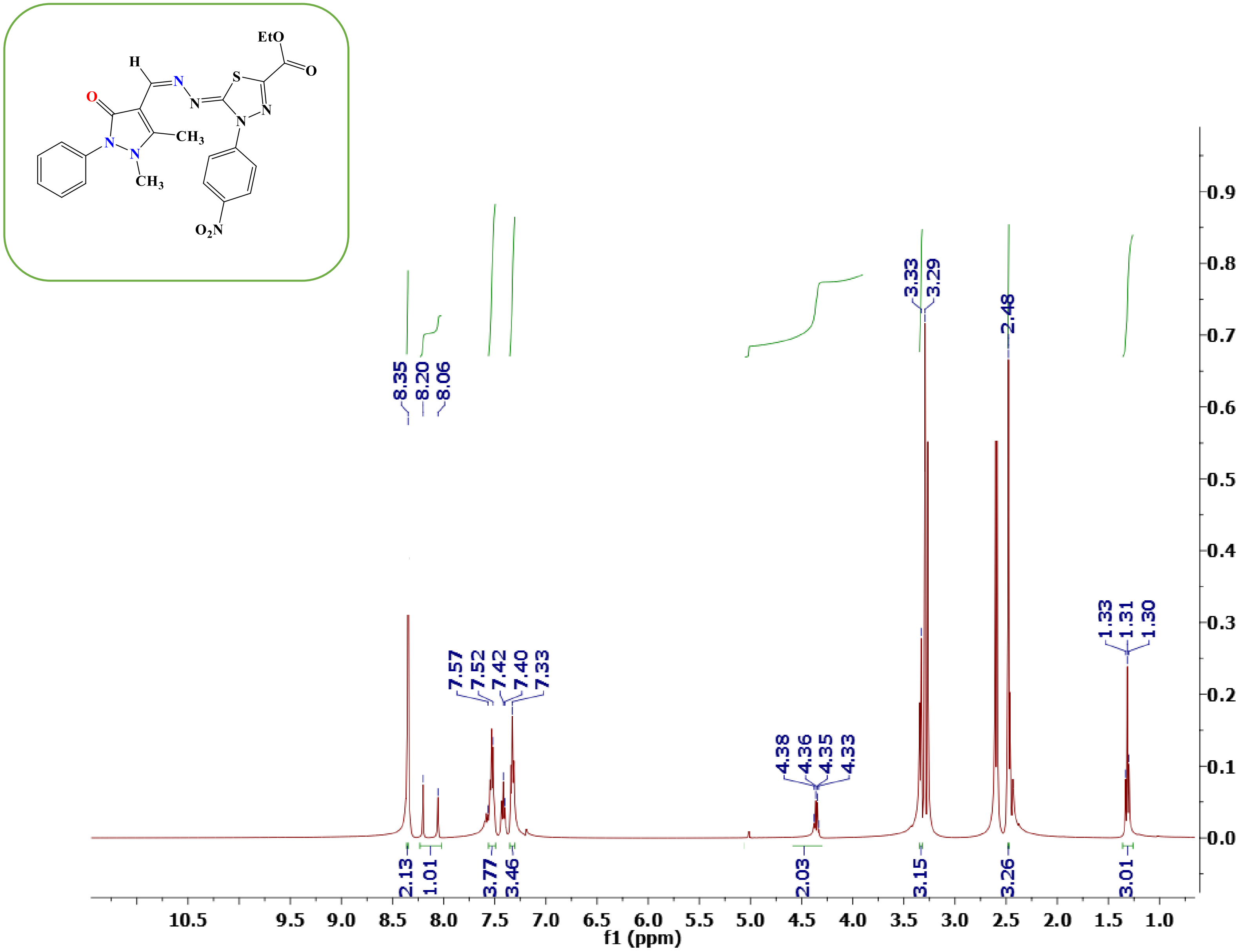

Figure S10: H^1-NMR spectrum of compound no.7

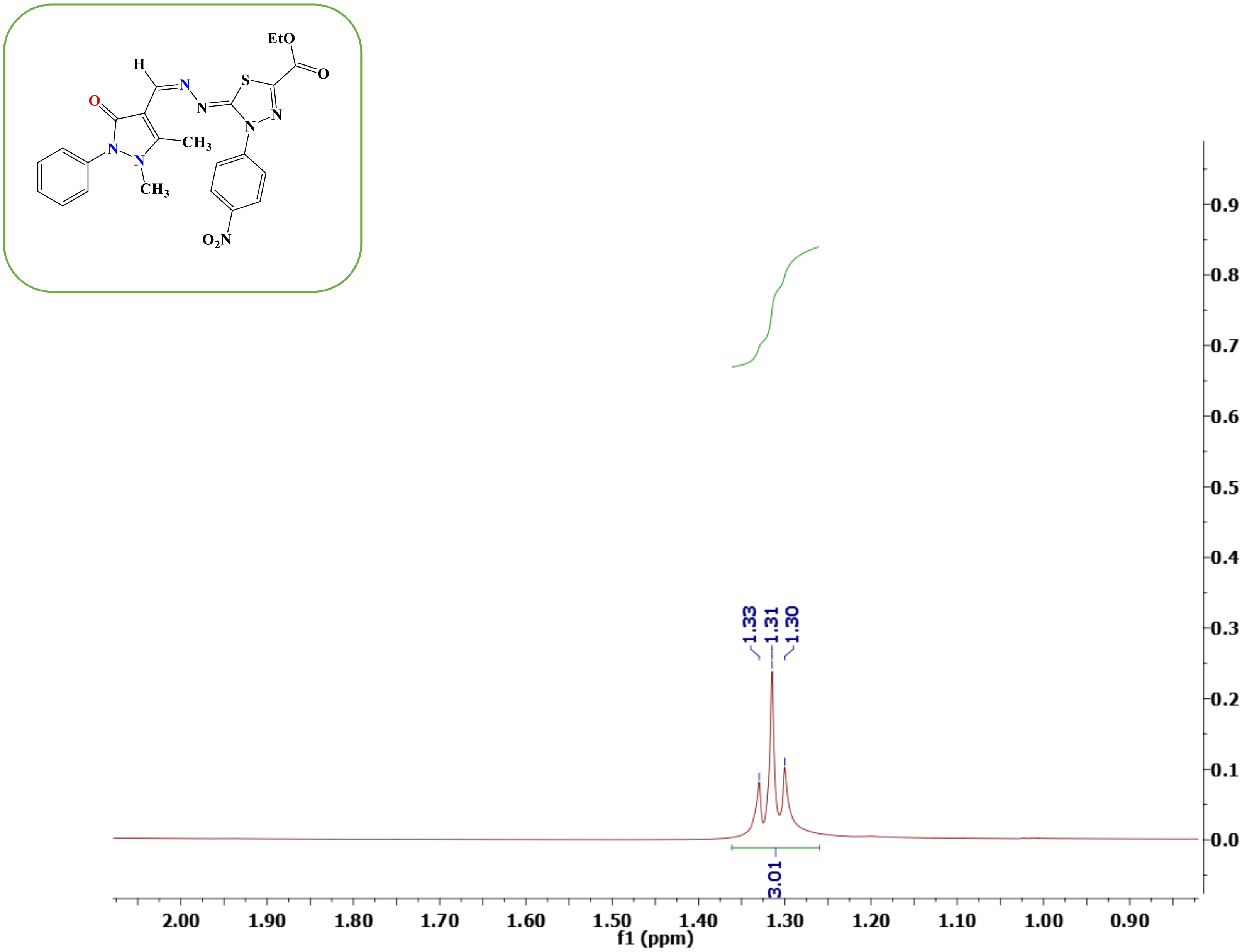

Figure S11: H[1]-NMR spectrum of compound no.7

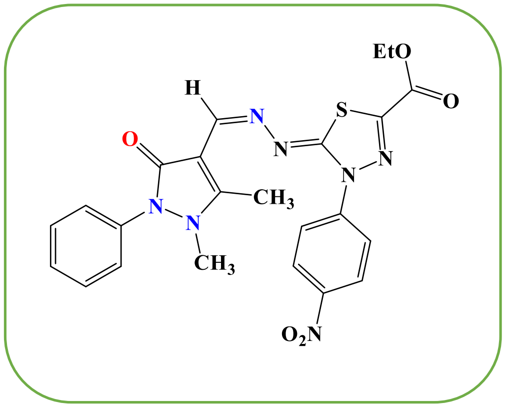

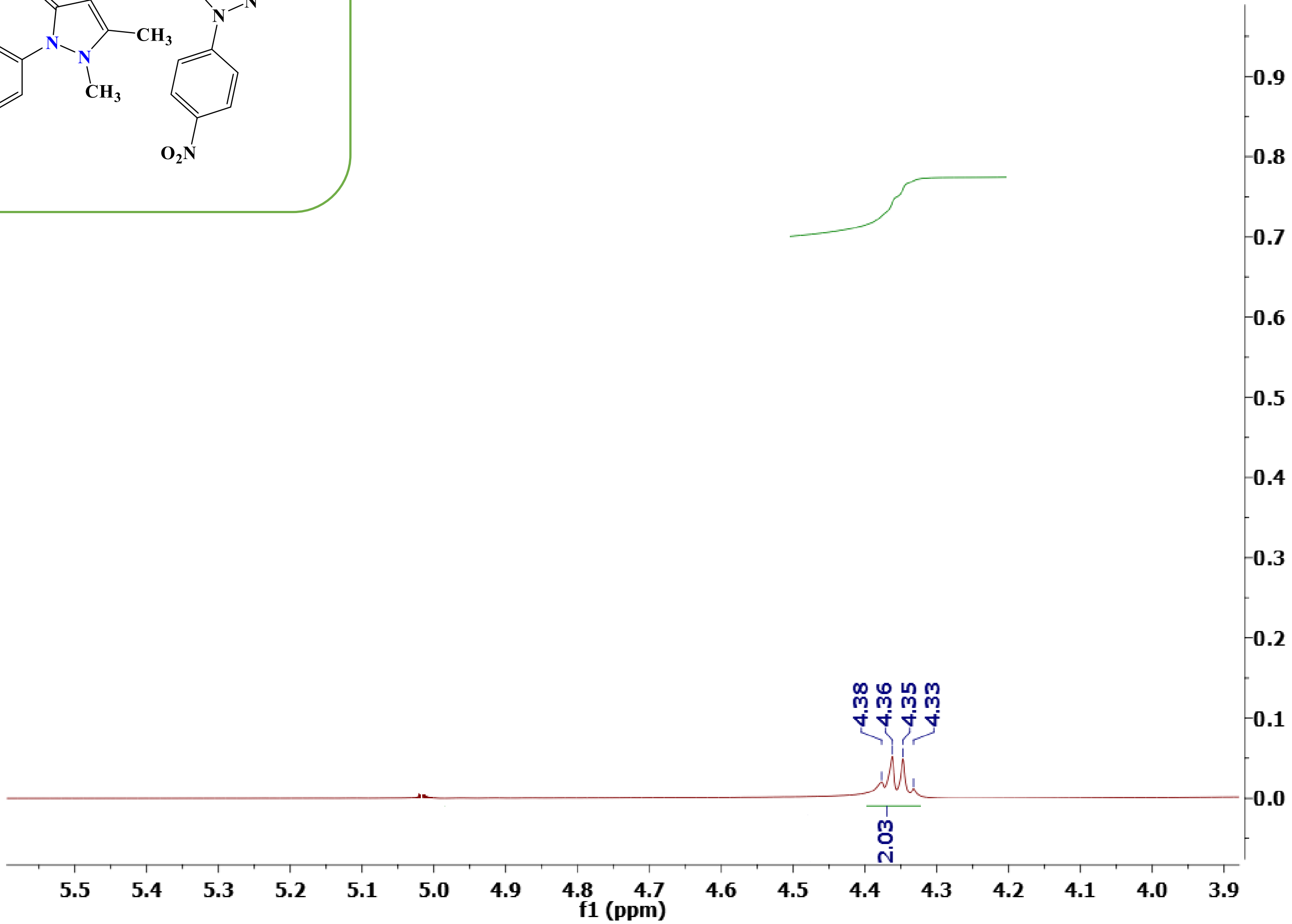

Figure S12: H^1-NMR spectrum of compound no.7

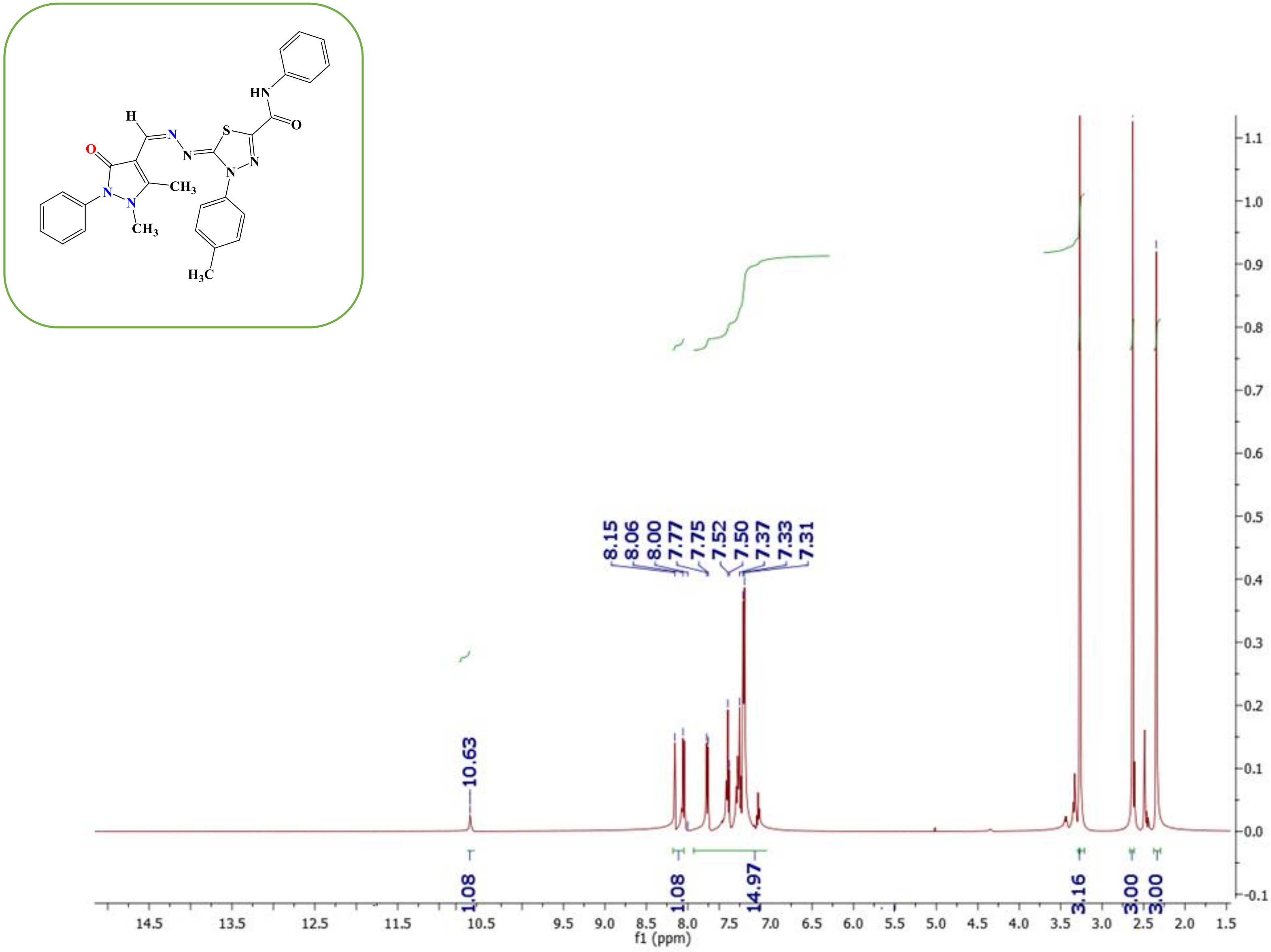

Figure S13: H¹-NMR spectrum of compound no.8

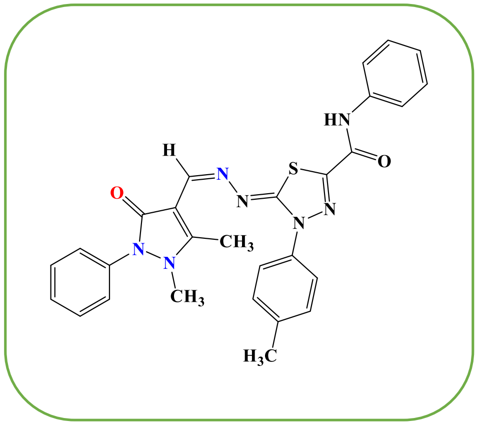

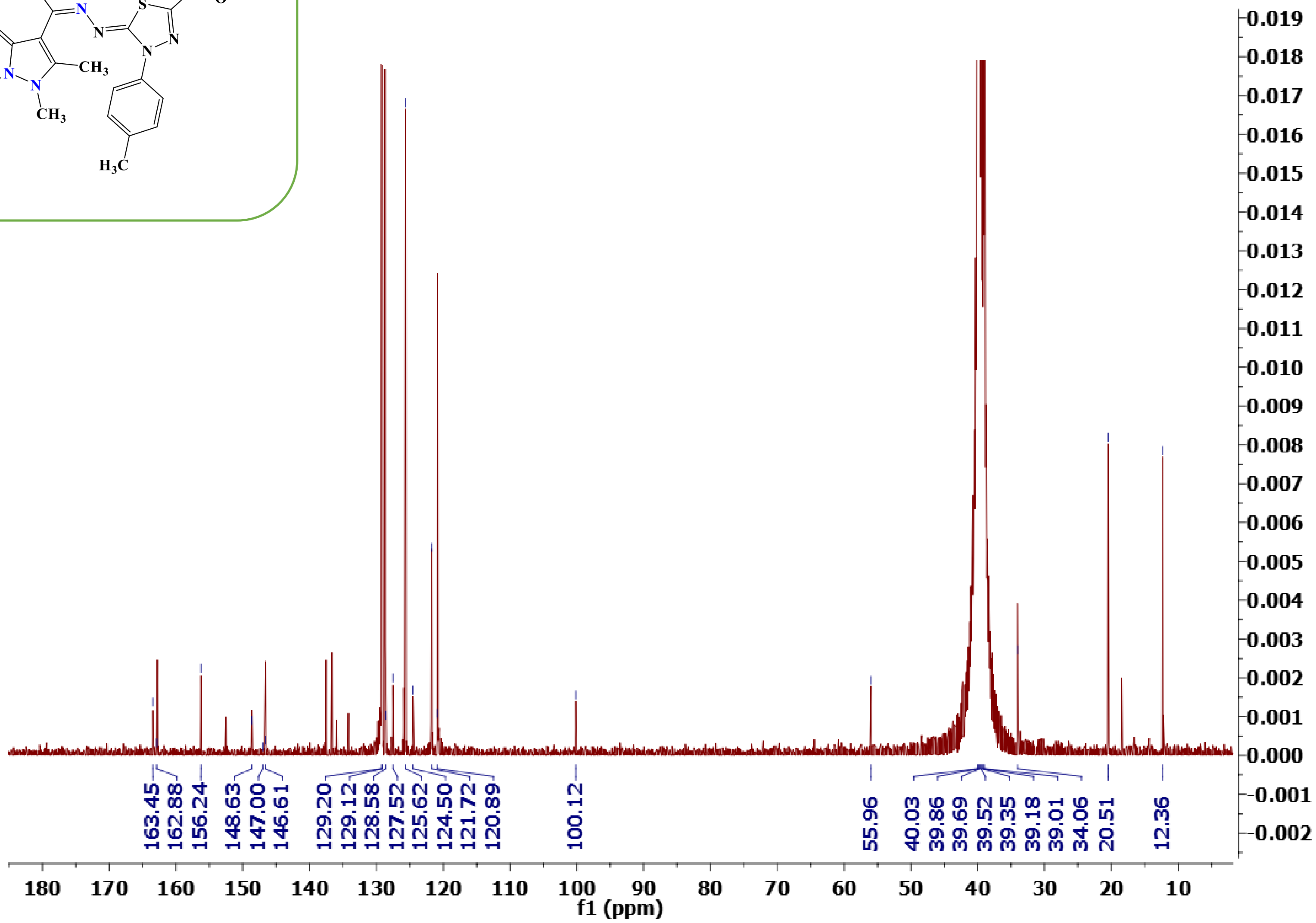

Figure S14: C13-NMR spectrum of compound no.8

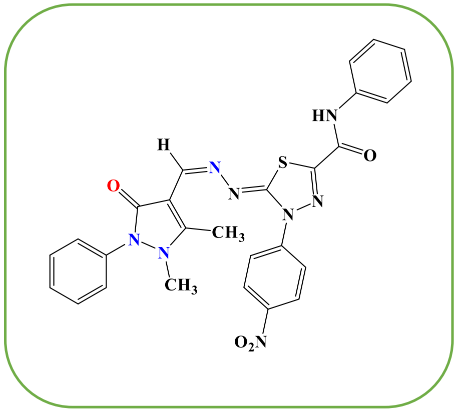

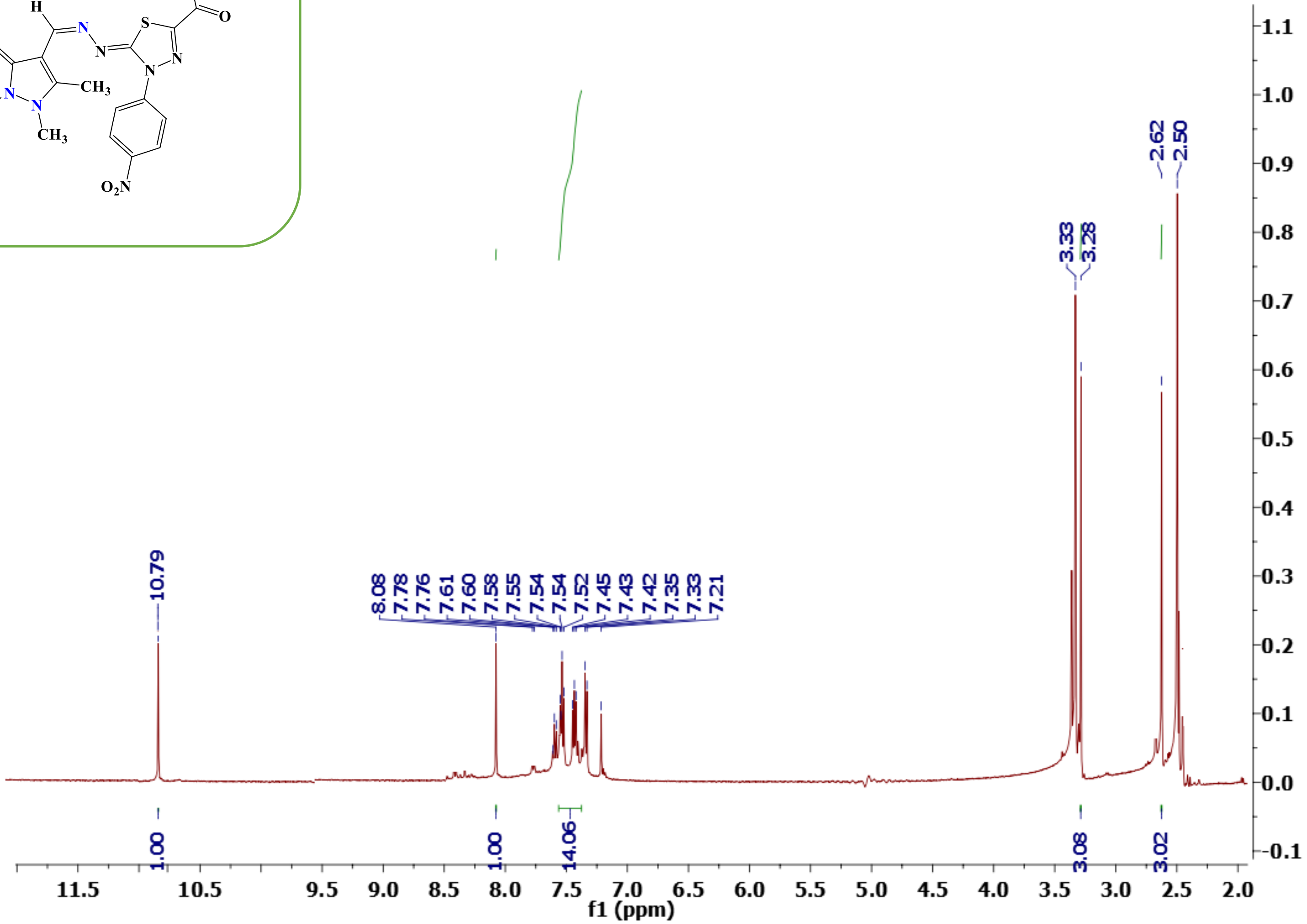

Figure S15:: H1-NMR spectrum of compound no.9

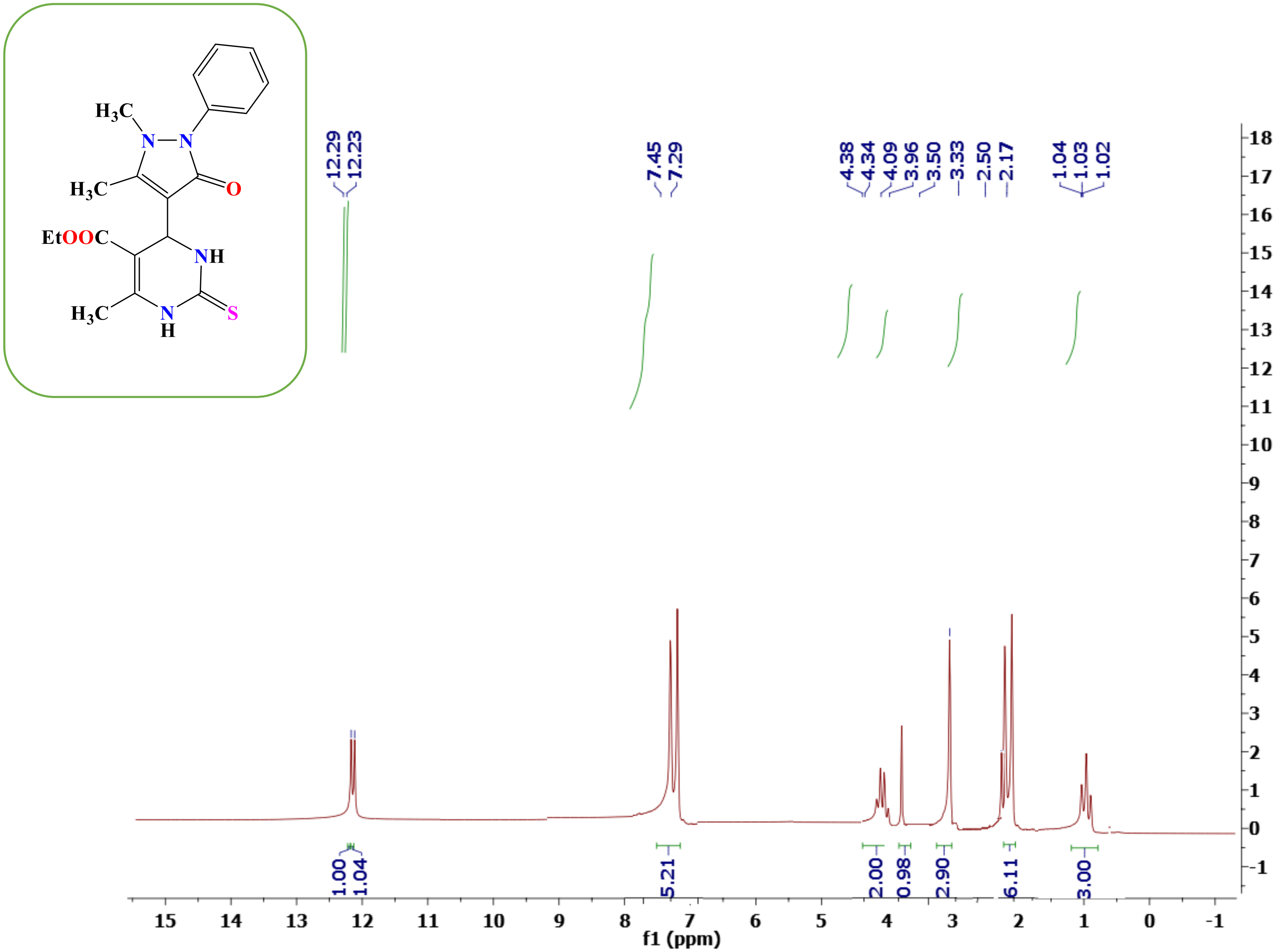

Figure S16: H1-NMR spectrum of compound no.11

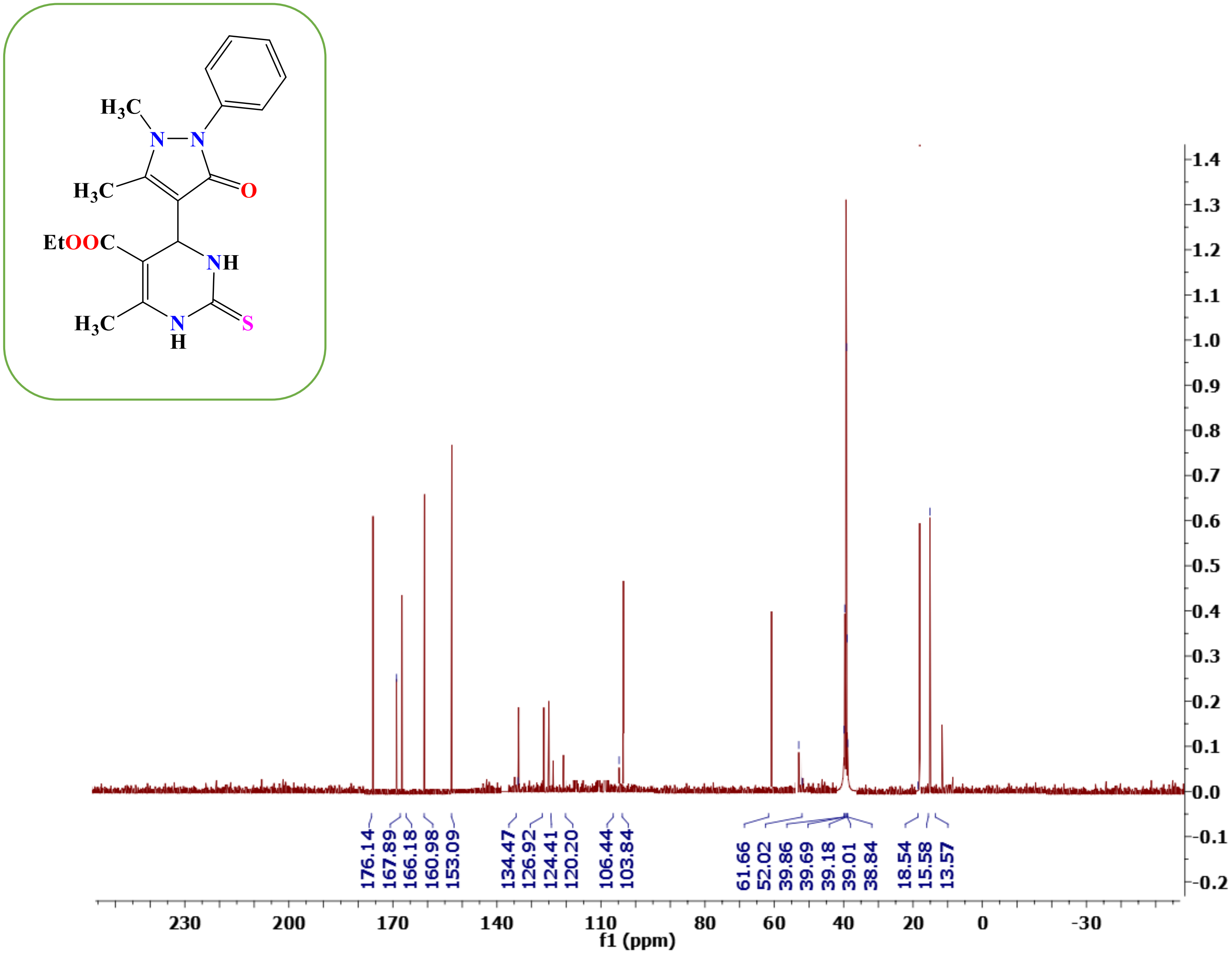

Figure S17: C[13]-NMR spectrum of compound no.11

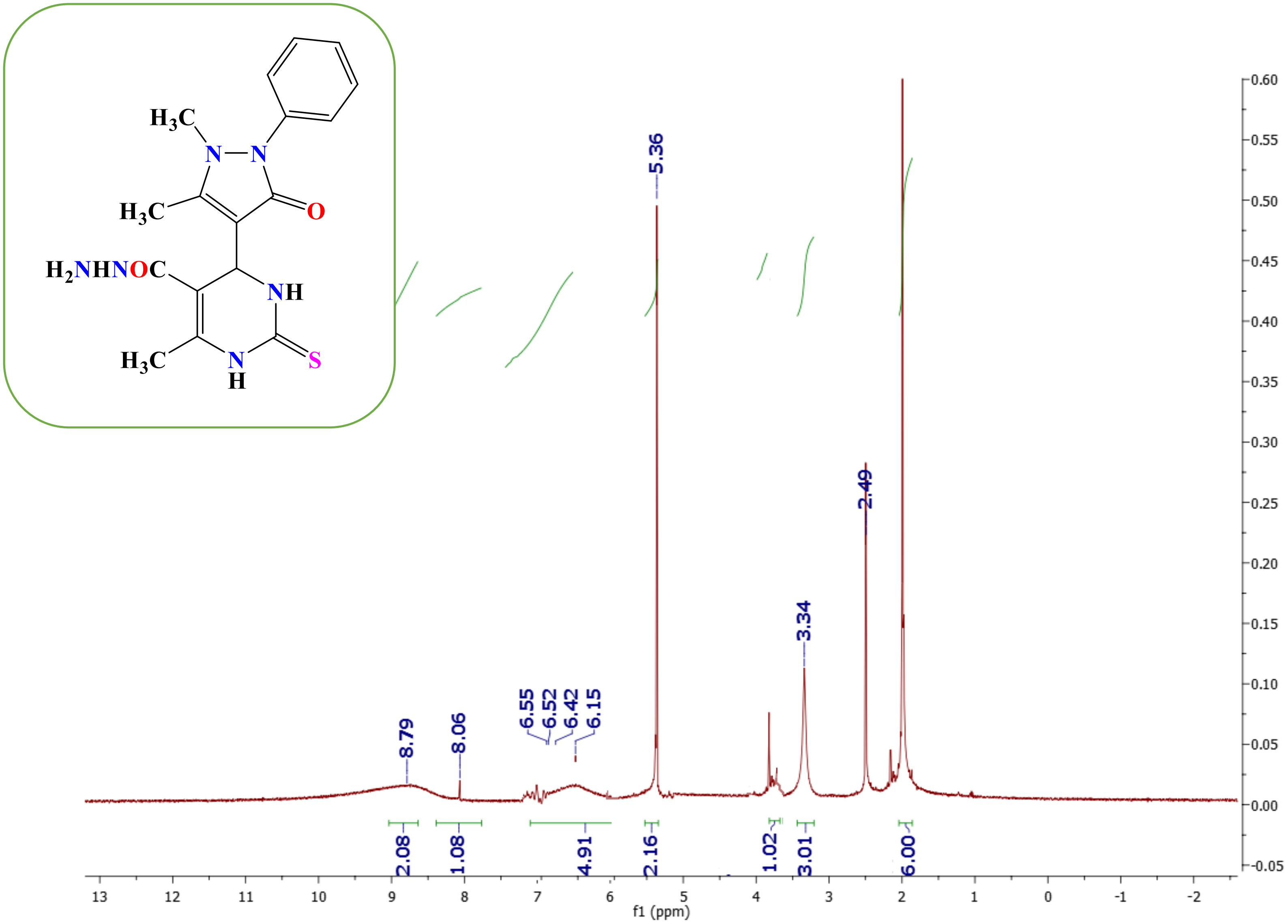

Figure S18: H1-NMR spectrum of compound no.12
